# A Comprehensive Insight into Tetracycline Resistant Bacteria and Antibiotic Resistance Genes in Activated Sludge Using Next-Generation Sequencing

**DOI:** 10.3390/ijms150610083

**Published:** 2014-06-05

**Authors:** Kailong Huang, Junying Tang, Xu-Xiang Zhang, Ke Xu, Hongqiang Ren

**Affiliations:** State Key Laboratory of Pollution Control and Resource Reuse, Environmental Health Research Center, School of the Environment, Nanjing University, Nanjing 210023, China; E-Mails: huangkailongnju@gmail.com (K.H.); tjynju@gmail.com (J.T.); kexu@nju.edu.cn (K.X.)

**Keywords:** antibiotic resistant bacteria, antibiotic resistance genes, sewage treatment plant, tetracycline, 454 pyrosequencing, metagenomic analysis

## Abstract

In order to comprehensively investigate tetracycline resistance in activated sludge of sewage treatment plants, 454 pyrosequencing and Illumina high-throughput sequencing were used to detect potential tetracycline resistant bacteria (TRB) and antibiotic resistance genes (ARGs) in sludge cultured with different concentrations of tetracycline. Pyrosequencing of 16S rRNA gene revealed that tetracycline treatment greatly affected the bacterial community structure of the sludge. Nine genera consisting of *Sulfuritalea*, *Armatimonas*, *Prosthecobacter*, *Hyphomicrobium*, *Azonexus*, *Longilinea*, *Paracoccus*, *Novosphingobium* and *Rhodobacter* were identified as potential TRB in the sludge. Results of qPCR, molecular cloning and metagenomic analysis consistently indicated that tetracycline treatment could increase both the abundance and diversity of the *tet* genes, but decreased the occurrence and diversity of non-tetracycline ARG, especially sulfonamide resistance gene *sul2*. Cluster analysis showed that tetracycline treatment at subinhibitory concentrations (5 mg/L) was found to pose greater effects on the bacterial community composition, which may be responsible for the variations of the ARGs abundance. This study indicated that joint use of 454 pyrosequencing and Illumina high-throughput sequencing can be effectively used to explore ARB and ARGs in the environment, and future studies should include an in-depth investigation of the relationship between microbial community, ARGs and antibiotics in sewage treatment plant (STP) sludge.

## 1. Introduction

Extensive use and abuse of antibiotics in health protection and agricultural production have led to the emergence of widespread various antibiotic resistance genes (ARGs) and resistant bacteria (ARB) in the environment [1,2], which is thought to pose an ever increasing threat to public health [3]. The broad spectrum tetracyclines are one of the most frequently used classes of antibiotics for protection of human and animal health [4]. Previous studies have shown that the concentrations of tetracycline in livestock wastewater [5,6] and municipal sewage [7,8] were 4.1~32.67 μg/L and 89.4~652.6 ng/L, respectively. Increasing evidence suggested that sewage treatment plants (STPs) serve as important reservoirs for environmental tetracycline resistant bacteria (TRB) and resistance genes (*tet*) [9,10,11].

Both culture-based [9,10,12] and culture-independent approaches [13] have been used to explore the TRB in STPs. Classical microbiological methodology relies on plate counting of coliforms, which makes the assessment results unrepresentative and biased. Currently, molecular methods used for exploring TRB in sludge include polymerase chain reaction-denaturing gradient gel electrophoresis (PCR-DGGE) [14], quantitative real time PCR (qPCR) [14], molecular cloning [11] and microarray [15], but the methods are time- and cost-consuming due to low throughput. Recently, growing evidence has shown that next-generation sequencing is a powerful metagenomic tool for comprehensive overview of microbial communities and/or functional genes in various environmental compartments, including soil [16], human gastrointestinal tract [17], sediments [18], and wastewater treatment plants [19].

In this study, we designed a batch experiment to culture STP sludge in filtered sewage fed with different concentrations of tetracycline to identify TRB community composition in the sludge and to evaluate the effect of tetracycline stress on the abundance and diversity of *tet* genes. 454 Pyrosequencing was used to explore the TRB in activated sludge based on PCR of bacterial 16S rRNA gene. Illumina high-throughput sequencing in combination with qPCR and molecular cloning were also employed to investigate the relative abundance and diversity of ARGs including *tet* genes. This study revealed the distribution patterns of TRB and ARGs in activated sludge and provided a useful tool for comprehensive investigation of tetracycline resistance in the environment.

## 2. Results and Discussion

### 2.1. Bacterial Community Shift under Tetracycline Stress

Pyrosequencing of 16S rRNA gene showed that 6-day tetracycline treatment separately at 1, 5 and 20 mg/L tended to increase the number of operational taxonomic units (OTUs) in the sludge, which agrees with the patterns of Chao 1 and Shannon index (Table 1). The reason may be that the growth of the dominant species in the sludge was inhibited under tetracycline stress, while more species with low abundance had the opportunity to survive and reproduce to reach the detection limit. Li *et al.* [20] also indicated that the antibiotic stresses seemed not effective in reduction of the bacterial diversities of river water. Interestingly, the sludge fed with 5 mg/L had the richest diversity (1692 OTUs), and 1 mg/L tetracycline treatment also increased the OTUs number, revealing that subinhibitory concentrations of tetracycline stress may favor enhancement of species richness [21].

**Table 1:** Number of 16S rRNA gene sequences analyzed, observed OTUs, Chao 1 and Shannon index for each sample at similarity of 97%.

Tetracycline Concentrations	No. of Raw Sequences	Observed OTUs	Chao 1	Shannon Index
0 mg/L	7097	1112	1562	5.94
1 mg/L	13,351	1306	1988	6.19
5 mg/L	9306	1692	2899	6.60
20 mg/L	12,802	1347	1975	6.35

OTUs: Operational taxonomic units; Chao 1: Chao 1 estimator.

**Figure 1:**
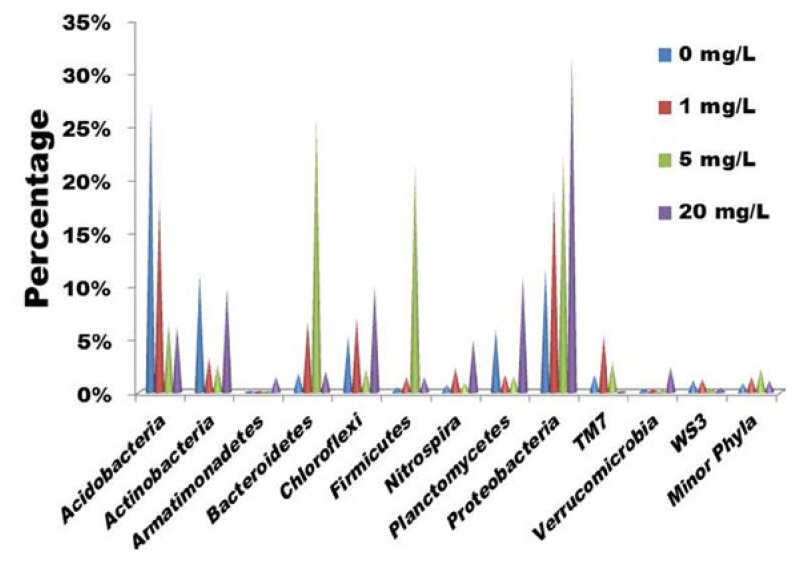
Abundance of various bacterial phyla in sludge after 6 days treatment with different concentrations of tetracycline (0~20 mg/L). The filtered pyrosequencing reads were classified using RDP Classifier at a confidence threshold of 80%. The relative abundance is presented as the percentage of each phylum in total effective reads of the corresponding sample.

As shown in Figure 1, Acidobacteria (27.3%) was the most abundant phylum in the sludge without tetracycline treatment, followed by Proteobacteria (11.6%), Actinobacteria (11.2%), Planctomycetes (5.9%), Chloroflexi (5.3%), Bacteroidetes (1.8%), TM 7 (1.7%), WS3 (1.1%), Nitrospira (0.7%) and Firmicutes (0.5%). Lozada *et al.* [22] also indicated that Proteobacteria and Acidobacteria were dominant in surfactant-enrichment lab-scale activated sludge. Acidobacteria, a common and predominant phylum in sludge [22], seems susceptible to tetracycline since the phylum abundance decreased from 27.3% under no tetracycline stress to 6.2% with 20 mg/L tetracycline treatment. Actinobacteria and Planctomycetes were also susceptible to tetracycline since their abundance evidently decreased with the increase of tetracycline concentration. On the contrary, tetracycline treatment dramatically increased the abundance of Proteobacteria in the sludge. Bacteriodetes and Firmicutes seemed to have higher abundance after 5 mg/L tetracycline treatment, but had lower abundance after 20 mg/L tetracycline treatment.

**Figure 2:**
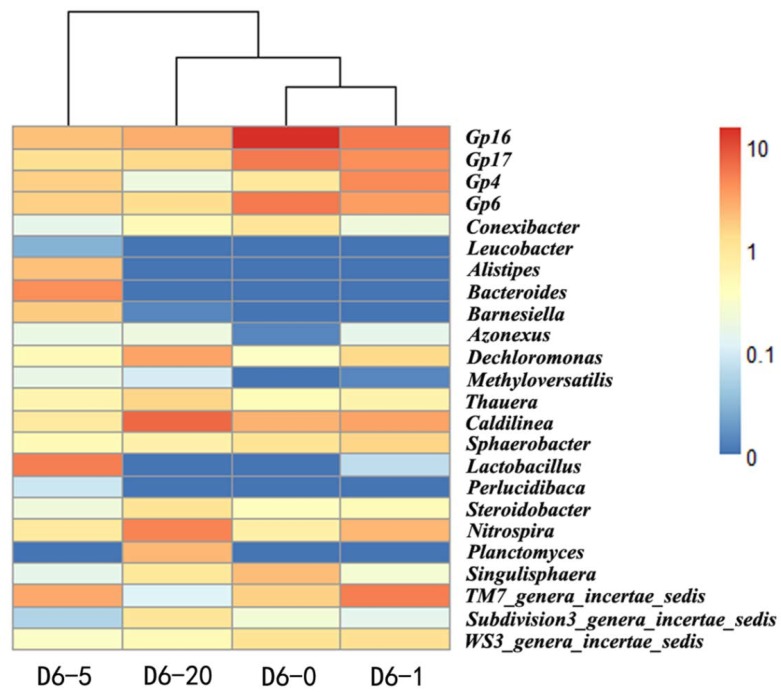
Heat map of genera occurring at >1% abundance in at least one sludge sample. Scale bar on the right shows the variation of the normalized abundance of the genera. D6-0, D6-1, D6-5 and D6-20: sludge cultured with 0, 1, 5 and 20 mg/L tetracycline for 6 days, respectively.

Figure 2 shows that at the level of genus, *Gp16* (14.9%) dominated in the sludge without tetracycline treatment, followed by *Gp17* (5.5%), *Gp6* (5.4%), *Caldilinea* (2.5%), *Singulisphaera* (2.1%), *TM7_genera_incertae_sedis* (1.7%), *Sphaerobacter* (1.1%), *WS3_genera_incertae_sedis* (1.1%) and *Conexibacter* (1.0%). Culture with 5 mg/L tetracycline decreased the abundance of *Gp16*, *Gp17*, *Gp6*, *Singulisphaera*, *Conexibacter and* *TM7_genera_incertae_sedis*. It should be noted that tetracycline treatment at subinhibitory concentrations (5 mg/L) considerably reduced *Acidobacteria* abundance. Within the *Acidobacteria* phylum, the *Gp16* genus was found very susceptible to tetracycline, since its abundance was 14.9%, 5.7% and 2.1% with tetracycline at 0, 1, and 5 mg/L, respectively (Figure 2). The subinhibitory-dose treatment tended to increase the abundance of *Bacteroidetes* and *Firmicutes* phyla (Figure 1), as well as *Azonexus*, *Methyloversatilis* and *Perlucidibaca* genera (Figure 2). Various bacterial strains of *Bacteroidetes*, *Firmicutes* and *Proteobacteria* have previously been isolated from livestock feces, farmyard manure and soil [23].

### 2.2. Identification of TRB in the Sludge

According to the National Antimicrobial Resistance Monitoring System, bacteria are identified as TRB if they can survive under 20 mg/L tetracycline stress [8,10,12]. In this study, the bacteria with successive increases of relative abundance in response of tetracycline stress enhancement were considered TRB.

In the sludge cultured with 20 mg/L tetracycline, TRB consisted of *Proteobacteria*, *Armatimonadetes*, *Verrucomicrobia* and *Chloroflexi* phyla, accounting for 60.61%, 16.97%, 15.76% and 6.67% of the total TRB community, respectively (Table 2). At the level of class, TRB consisted of *Betaproteobacteria*, *Alphaproteobacteria*, *Armatimonadia*, *Verrucomicrobiae* and *Anaerolineae*, among which *Betaproteobacteria* and *Alphaproteobacteria* were the main classes. This is supported by a previous study indicating that *Alphaproteobacteria* and *Betaproteobacteria* dominated in an oxytetracycline production wastewater treatment plant [24] and an aerobic reactor treating high-concentration antibiotic wastewater [25]. A total of nine genera were identified for TRB in the sludge, among which *Sulfuritalea* (0.54%) had the highest abundance, followed by *Armatimonas* (0.39%), *Prosthecobacter* (0.37%), *Hyphomicrobium* (0.34%), *Azonexus* (0.20%), *Longilinea* (0.15%), *Novosphingobium* (0.13%), *Paracoccus* (0.11%) and *Rhodobacter* (0.10%) (Table 2).

**Table 2:** Taxon composition profile of tetracycline resistant bacteria (TRB) in activated sludge.

Phylum	Class	Genus	D6-0	D6-1	D6-5	D6-20
*Proteobacteria*	*Betaproteobacteria*	*Sulfuritalea*	0.01%	0.10%	0.10%	0.54%
*Armatimonadetes*	*Armatimonadia*	*Armatimonas*	ND	ND	0.01%	0.39%
*Verrucomicrobia*	*Verrucomicrobiae*	*Prosthecobacter*	ND	ND	0.01%	0.37%
*Proteobacteria*	*Alphaproteobacteria*	*Hyphomicrobium*	ND	0.11%	0.13%	0.34%
*Proteobacteria*	*Betaproteobacteria*	*Azonexus*	0.01%	0.15%	0.18%	0.20%
*Chloroflexi*	*Anaerolineae*	*Longilinea*	ND	0.01%	0.01%	0.15%
*Proteobacteria*	*Alphaproteobacteria*	*Novosphingobium*	0.04%	0.04%	0.07%	0.13%
*Proteobacteria*	*Alphaproteobacteria*	*Paracoccus*	ND	0.01%	0.01%	0.11%
*Proteobacteria*	*Alphaproteobacteria*	*Rhodobacter*	ND	0.01%	0.03%	0.10%

ND: Not detectable; D6-0, D6-1, D6-5 and D6-20: sludge cultured with 0, 1, 5 and 20 mg/L tetracycline for 6 days, respectively.

*Paracoccus* serving as an important denitrifier [26] has been reported to be TRB in STP sludge treated with tetracycline [13]. To our knowledge, the other 8 genera were firstly identified as TRB in this study, indicating that pyrosequencing is a new powerful tool to profile the ARB communities in the environment. An in-depth investigation showed that the function of the newly identified TRB mainly included denitrification and degradation. The TRB genus *Sulfuritalea* dominating in the sludge is a denitrifier frequently detected in freshwater lakes [27]. Both nitrate-reduction bacterium *Azonexus caeni* [28] and denitrifying photosynthetic bacteria *Rhodobacter* [29] were previously isolated from sludge of wastewater treatment plants. *Hyphomicrobium* sp. can grow on media with chloromethane, methanol, methylamine and ethanol as sole carbon and energy sources, and the microorganism has been used for bioremediation of gasoline-contaminated site [30]. *Novosphingobium* sp. is widely distributed in the environment, e.g., groundwater treatment bioreactor [31], deep-sea environment [32] and freshwater lakes [33], and can degrade various aromatic compounds including polychlorophenol and polycyclic aromatic hydrocarbons. In addition, it has been reported that *Novosphingobium* sp. isolated from activated sludge of a Japanese STP is capable of estradiol degradation [34].

### 2.3. Effects of Tetracycline Stress on the Abundance and Diversity of ARGs

**Figure 3:**
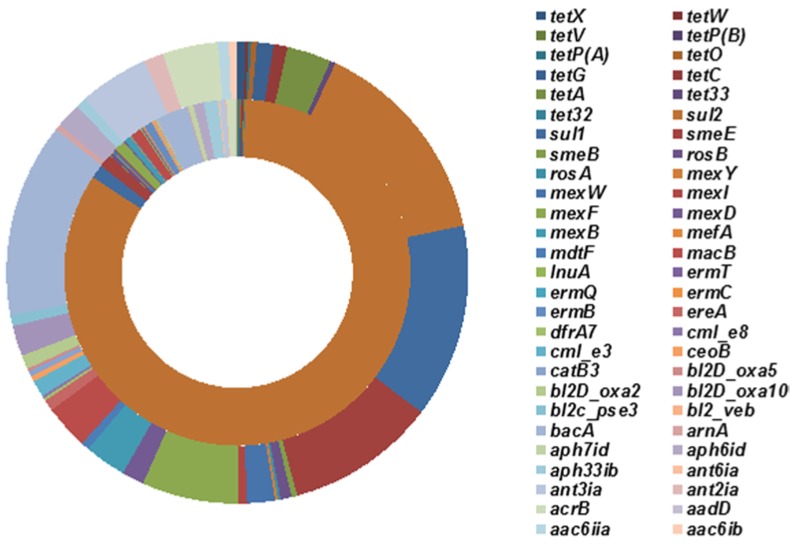
Relative abundance of antibiotic resistance genes (ARGs) in sludge of D6-0 (Inner ring) and D6-20 (Outer ring). After searching in antibiotic resistance database (ARDB), the relative abundance was obtained with the matched sequencing reads normalized to the total reads of each sample. D6-0: sludge incubated with 0 mg/L tetracycline for 6 days; D6-20: sludge incubated with 20 mg/L tetracycline for 6 days.

To investigate the impact of tetracycline stress on the abundance of ARGs, the sludge samples fed with 0 and 20 mg/L tetracycline were selected for Illumina high-throughput sequencing. Alignments of the Illumina reads against the Antibiotic Resistance Genes Database (ARDB) showed that a total of 2168 reads (0.0192%) from the sludge under no tetracycline stress and 515 reads (0.0043%) from the sludge fed with 20 mg/L tetracycline were annotated as 47 and 41 types of known ARGs, respectively (Figure 3). As a common hypothesis, ARGs may have higher abundance in the presence of antibiotics [35].

**Figure 4:**
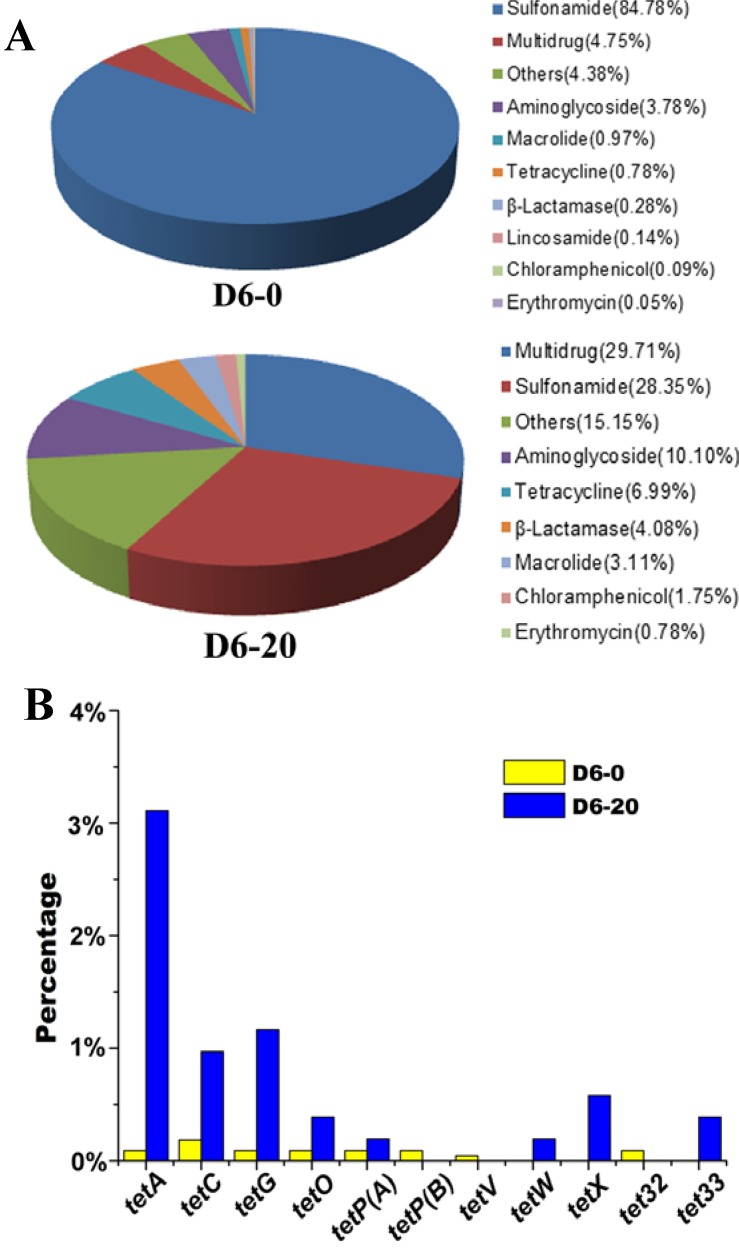
Antibiotic resistance patterns (**A**) and *tet* genes (**B**) in sludge treated with 0 mg/L (D6-0) and 20 mg/L (D6-20) tetracycline. The resistance genes identified were grouped according to antibiotic types after alignment of the high-throughput sequencing reads against antibiotic resistance database (ARDB). D6-0: sludge incubated with 0 mg/L tetracycline for 6 days; D6-20: sludge incubated with 20 mg/L tetracycline for 6 days.

However, this study showed that tetracycline treatment decreased both the occurrence and diversity of non-tetracycline ARGs, although the abundance of *tet* genes increased. The considerable decrease in the abundance of sulfonamide resistance gene *sul2* (from 83.49% to 14.76%) mainly contributed to the diminishment of the non-tetracycline ARGs (Figure 3). Table S1 shows that *sul2* (ARDB accession number: CAE53425) dominated in the sludge without tetracycline stress, but had much lower abundance in the sludge fed with 20 mg/L tetracycline (Table S2). *Pasteurella multocida* was often found to carry *sul*2 (http://ardb.cbcb.umd.edu/cgi/search.cgi?db=L&field=ni&term=CAE53425), and its growth can be inhibited by tetracycline via interference with protein synthesis by binding to the bacterial 30S ribosomal subunit [36]. Interestingly, this study showed that *Salmonella enterica* plasmid pCVM19633_110 and *Pasteurella multocida* plasmid pCCK381 predominant in the sludge under no tetracycline stress (Table S3) had much lower abundance in the sludge fed with 20 mg/L tetracycline (Table S4). This may be also responsible for the reduction of *sul2* abundance induced by tetracycline treatment since *sul2* are located on the genomes of the two plasmids [35]. In addition, we summarized the types of the ARGs detected in the sludge separately fed with 0 and 20 mg/L tetracycline (Figure 4A). Most of the assigned sequencing reads were found to be involved in sulfonamide resistance in the sludge containing no tetracycline (84.78%) and the sludge fed with 20 mg/L tetracycline (28.35%). Figure 4A illustrates that the tetracycline selection pressure (20 mg/L) promoted multidrug, aminoglycoside and tetracycline (from 0.78% to 6.99%) resistances in the sludge. A previous study also indicated that incubation in the presence of tetracycline favored the emergence of multidrug-resistance mutants in *Pseudomonas aeruginosa* [37].

**Figure 5:**
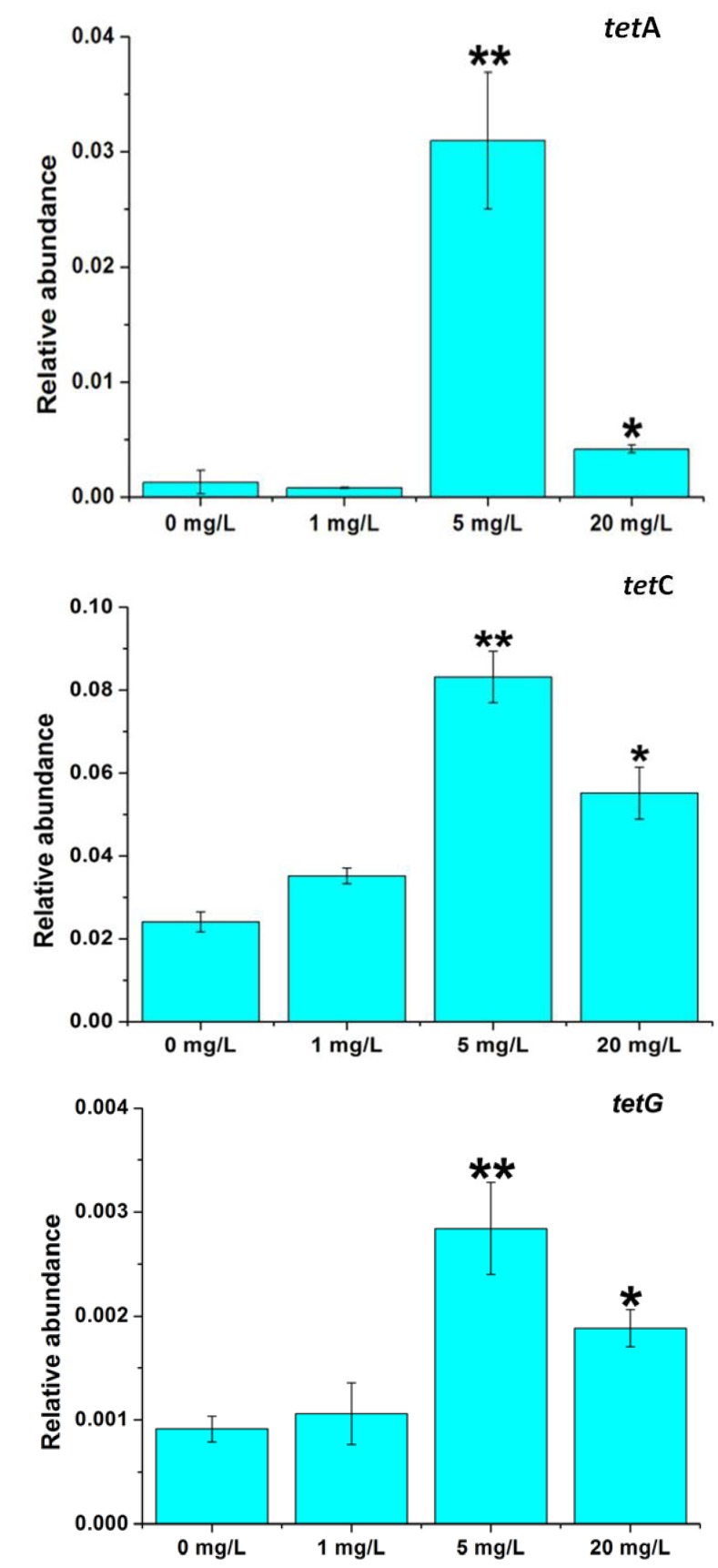
Relative abundance of *tetA*, *tetC* and *tetG* in sludge fed with different concentrations of tetracycline for 6 days. qPCR was used to determine the relative abundance normalized to the total copy number of 16S rRNA genes in corresponding samples. ** *p* < 0.01, comparing 5 mg/L with 0 mg/L; * *p* < 0.05, comparing 20 mg/L with 0 mg/L.

PCRs showed that 11 *tet* genes among the 15 tested genes were present in the sludge, including *tetA*, *tetB*, *tetC*, *tetG*, *tetK* and *tetP(A)* encoding tetracycline efflux proteins, *tetM*, *tetO*, *tetS* and *tetW* encoding ribosomal protection proteins, and *tetX* encoding enzymatic modification protein (Figure S1). Previous studies showed that *tetA*, *tetC* and *tetG* were more abundant than other *tet* genes [11,38], so *tetA*, *tetC* and *tetG* were selected for qPCR to investigate the impact of tetracycline stress on the abundance of tet genes. *TetC* had higher abundance than *tetA* and *tetG* by an order of magnitude, which indicated that *tetC* might play an important role in tetracycline resistance in the STP sludge. After 6 days treatment, the sludge fed with 5 or 20 mg/L tetracycline had comparatively higher levels of *tetA*, *tetC* and *tetG*, but 1 mg/L tetracycline treatment posed no evident effect (*p* > 0.05) (Figure 5). The result is confirmed by metagenomic analysis showing that *tetA*, *tetC* and *tetG* genes increased from 0.09% to 3.11%, 0.18% to 0.97% and 0.09% to 1.17% after 6 days treatment with 20 mg/L tetracycline, respectively (Figure 4B). Enhancement of tetracycline concentration led to significant increase in the relative abundance of *tetA*, *tetC* and *tetG* (Figure 4B and Figure 5), which may result from the microbial community shift (Figure 2). Li *et al*. [13] reported a similar result that *tetA* and *tetG* significantly increased after tetracycline treatment. In this study, metagenomic analysis also indicated that the relative abundance of *tetO*, *tetP(A)*, *tetW*, *tetX* and *tet33* increased after tetracycline incubation, but some minor tet genes, e.g., *tetP(B)*, *tetV* and *tet32*, had lower abundance in response of tetracycline treatment (Figure 4B). Zhang *et al*. [14] indicated that proliferation of the ARGs can be accelerated in the activated sludge under tetracycline pressure. qPCR results showed that the tet genes tended to have the highest abundance under the condition of 5 mg/L tetracycline (Figure 5), which is confirmed by the pyrosequencing results demonstrating that the bacterial community structure of the sludge treated with 5 mg/L tetracycline were evidently divergent from those of the other three sludge samples (Figure 2). Further, it is known that antibiotic treatment at subinhibitory concentrations can increase the rate of mutation, horizontal gene transfer and spread of antibiotic resistance [39].

Most of the functional genes are considered conserved, but a previous study [11] showed that *tetG* had an extremely high diversity in STPs. In this study, a total of 52 clones, including 26 clones from the sludge containing no tetracycline and 26 clones from the sludge incubated with 20 mg/L tetracycline, were selected to investigate the effect of tetracycline on diversity of *tetG*. Results showed that 19 *tetG* genotypes occurred in the sludge under no tetracycline stress and 21 genotypes were present in the sludge treated with 20 mg/L tetracycline.

**Figure 6:**
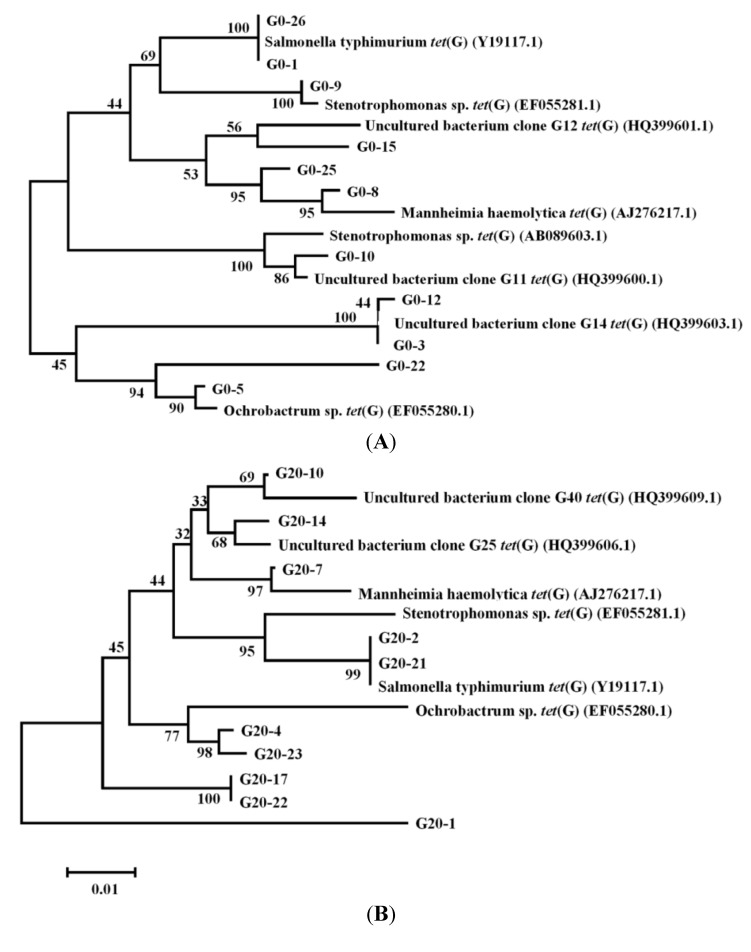
Neighbor-joining phylogenetic analysis of *tetG* diversity in activated sludge of D6-0 (**A**) and D6-20 (**B**). The tree was constructed using MEGA version 5.05 and bootstrap analysis with 1000 replicates was used to evaluate the significance of the nodes. D6-0: sludge fed with 0 mg/L tetracycline for 6 days; D6-20: sludge fed with 20 mg/L tetracycline for 6 days.

Among the clones of sludge fed with no tetracycline, types G0-1 and G0-26 had a 100% identity to *Salmonella typhimurium* *tetG* (Y19117.1), and G0-9 were identified as the corresponding sequence of *Stenotrophomonas* sp. *tetG* (EF055281.1). Each of the types G0-8 and G0-25 had a similarity of 95% to the most closely related known gene: *Mannheimia haemolytica* *tetG* (AJ276217.1), while G0-5 and G0-22 had a sequence identity of only 94% to *Ochrobactrum* sp. *tetG* (EF055280.1) (Figure 6A). Tetracycline may increase the diversity of *tetG*, since five genotypes of *tetG* cloned from the sludge treated with 20 mg/L tetracycline could not be matched to the known *tetG* genes deposited in GenBank (Figure 6B). The selective pressure resulted from absorption of tetracycline by activated sludge may contribute to alterations on *tetG* DNA sequences [40].

## 3. Materials and Methods

### 3.1. Batch Experiments

Untreated sewage wastewater (25 L) and activated sludge (5 L) were sampled from Jiangxinzhou STP (Nanjing, China). Water and sludge samples were transported on ice to lab within 2 h. Sewage was filtered by 0.45 μm nitrate cellulose membrane. Activated sludge was centrifuged at 4000 rpm for 10 min under 4 °C, and the pellets were dissolved in 1 L of the filtered sewage water. In the batch assay, four 500 mL glass flasks with activated sludge mixed liquor (300 mL) separately containing 0, 1, 5 and 20 mg/L tetracycline [8,10,12,13] were run continuously at (26 ± 1) °C for 6 days. The glass flasks were cultured in a shaking incubator at 140 rpm for the aeration and mixing, and the incubator was covered with aluminum foil to avoid possible tetracycline photolysis. Every 24 h for 6 days, one third (100 mL, *v*/*v*) of the sludge samples were taken out from the reactors for biomass determination and DNA extraction. The remaining mixed liquor (200 mL) was centrifuged at 4000 rpm for 10 min under 4 °C. The pellets were transferred back to the reactors and then re-suspended with 300 mL filtered sewage containing corresponding concentrations of tetracycline (0, 1, 5 or 20 mg/L) for subsequent 24-h culture.

### 3.2. DNA Extraction and PCR

For DNA protection, activated sludge was sampled from the reactors every 24 h and mixed with 100% ethanol immediately at a ratio of 1:1 (*v*/*v*). The mixture was centrifuged at 4000 rpm for 10 min under 4 °C to collect the pellets (approximately 200 mg) for DNA extraction. The total DNA extraction was conducted using the FastDNA SPIN Kit for Soil (MP Biomedicals, Santa Ana, CA, USA). The DNA concentrations and purity were determined through microspectrophotometry (NanoDrop^®^ND-2000, NanoDrop Technologies, Willmington, DE, USA). The DNA products were stored at −20 °C until further molecular analyses.

According to the previous studies, 15 *tet* genes were amplified using primers listed in Table S5 in a 30-μL reaction mixture containing 1× PCR buffer, 100 μM dNTP, 2 pmol of each primer, 150 ng of template DNA and 1 U of EX Taq polymerase (TaKaRa, Shiga, Japan). *TetO* and *tetS* were amplified using the following conditions: initial denaturation at 95 °C for 7 min, followed by 40 cycles of 94 °C for 15 s, 50.3 °C (*tetO*) or 56 °C (*tetS*) for 30 s and 72 °C for 30 s, with a final extension of 72 °C for 7 min. PCR of *tetW* was carried out through: initial denaturation at 94 °C for 5 min, followed by 25 cycles of 94 °C for 30 s, 64 °C for 30 s and 72 °C for 30 s, with a final extension of 72 °C for 7 min. For the other 12 *tet* genes, PCR amplification was conducted according to the following protocols: initial denaturation at 94 °C for 5 min, followed by 35 cycles of denaturation at 94 °C for 1 min, annealing for 1 min at different temperatures (Table S5) and extension at 72 °C for 1.5 min, with a final elongation step at 72 °C for 10 min. The PCR products obtained were analyzed by gel electrophoresis using 1% (*w*/*v*) agarose in 1× TAE buffer and further confirmed by DNA sequencing. To check reproducibility, duplicate PCR reactions were performed for each sample and sterile water was used as the negative control. The PCR products (longer than 200 bp) sequencing data were deposited in NCBI under accession number KJ603161~KJ603167.

### 3.3. Quantitative Real-Time PCR

*TetA*, *tetC* and *tetG* were selected for quantitative assay using SYBR Green I qPCR. The plasmids containing target genes were obtained by molecular cloning. In detail, the PCR products of each *tet* gene were purified using the DNA Fragment Purification Kit (TaKaRa, Shiga, Japan) and cloned into the pMD19-T Vector (TaKaRa, Shiga, Japan). Plasmids carrying each *tet* gene were extracted and purified using the MiniBest Plasmid Purification Kit (TaKaRa, Shiga, Japan). Plasmid concentrations were determined by NanoDrop^®^ND-2000 (NanoDrop Technologies, Willmington, DE, USA). qPCRs were performed in 96-well plates with a final volume of 20 μL containing SYBR Premix EX Taq (TaKaRa, Shiga, Japan) super mix (10 μL), 10 μM primer (0.2 μL each), DNA templates (8 μL) and ddH_2_O (1.6 μL). Thermal cycling and fluorescence detection were conducted in Corbett Real-Time PCR with the Rotor-Gene 6000 Series Software 1.7 (QIAGEN, Nijmegen Area, The Netherlands). qPCR were performed using the following protocol: 94 °C for 3 min, followed by 40 cycles of 94 °C for 30 s, annealing at different temperatures (Table S5) for 45 s, and extension at 72 °C for 45 s. Each reaction was run in triplicate.

Five to seven-point calibration curves (*C*_t_ value *versus* log of initial *tet* gene copy) were generated for qPCR using 10-fold serial dilution of the *tet*-carrying plasmids. The PCR efficiency of each gene ranged from 92.1% to 102.6% with *R*^2^ values more than 0.995 for all calibration curves. Based on the calibration curves, the abundance of *tet* genes were calculated through the *C*_t_ values of the experimental samples. To minimize the potential variations in extraction efficiencies, eubacterial 16S rRNA genes were quantified using the method recommended by López-Gutiérrez *et al.* [41], and the relative abundance of *tet* genes was normalized to the total bacterial community.

### 3.4. Cloning and Phylogenetic Analysis of tetG

The PCR products of *tetG* were cloned to investigate the diversity of the genes in the sludge treated with 0 and 20 mg/L for 6 days. The purified PCR products were cloned to pMD19-T Vector (TaKaRa, Shiga, Japan). A total of 52 clones were randomly selected for the library construction, sequencing and subsequent similarity analysis. Nucleotide sequences of *tetG* were aligned using CLUSTALW [42]. The clones sharing a consensus sequence were grouped into one genotype, and only one representative in each group was selected for construction of phylogenetic trees. The GenBank sequences having the highest identity to the sequences obtained in this study were retrieved for phylogenetic trees construction. The neighbor-joining trees were constructed using Molecular Evolutionary Genetics Analysis (MEGA version 5.05) [42] and bootstrap analysis with 1000 replicates was conducted to evaluate the significance of the nodes. The 52 sequences of *tetG* cloning obtained in this study have been deposited in NCBI (Accession number: KJ603168~KJ603219).

### 3.5. 454 Pyrosequencing

The DNA extracted from activated sludge dosed with different levels of tetracycline (0, 1, 5 and 20 mg/L) for 6 days were subjected to Beijing Genome Institute (Shenzhen, China) for 16S rRNA gene pyrosequencing. The primers, V3F (5'-ACTCCTACGGGAGGCAGCAG-3') and V4R (5'-TACNVGGGTATCTAATCC-3'), targeting the hypervariable V3–V4 region (about 460 bp) were used to amplify the bacterial 16S rRNA gene. PCRs were conducted in a reaction system (50 μL) containing 1× Pfx Amplification Buffer (Invitrogen, Carlsbad, CA, USA), 0.4 mM dNTP, 2 mM MgSO_4_, 0.4 μM each fusion primer, 1 μL of template DNA and 2 U of Platinum^®^ Pfx DNA Polymerase (Invitrogen, Carlsbad, CA, USA). The 10 nucleotide “barcode” was permuted for each sample to separate the corresponding reads from the data pool generated in a single pyrosequencing run. PCR amplification was performed according to the following protocols: initial denaturation at 94 °C for 3 min, followed by 30 cycles of 94 °C for 30 s, 62 °C for 30 s and 70 °C for 45 s, with a final elongation step at 70 °C for 7 min. In order to minimize the impact of potential early-round PCR errors, amplicon libraries were prepared by a cocktail of three independent PCR products for each sample. The PCR products were purified using QIAquick PCR Purification Kit (Qiagen, Hilden, Germany) and quantified with an Agilent 2100 Bioanalyzer (Agilnet, Santa Clara, CA, USA). Equal DNA mass of each purified amplicon library from different samples were mixed for pyrosequencing on the Roche 454 FLX Titanium platform (Roche, Indianapolis, IN, USA) at Beijing Genome Institute (Shenzhen, China). The sequencing data were deposited in NCBI Sequence Read Archive under accession number SRP035342.

### 3.6. Illumina High-Throughput Sequencing

The metagenomic DNA extracted from the sludge cultured with 0 and 20 mg/L tetracycline was individually subjected to high-throughput sequencing using Illumina Hiseq 2000 (Illumina, San Diego, CA, USA) according to the manufacturer’s instructions. The “Index 101 PE” (Paired End sequencing, 101-bp reads and 8-bp index sequence) sequencing strategy was used for the high-throughput sequencing, which generates nearly equal amount of clean reads for each sample. A base-calling pipeline (Sequencing Control Software, Illumina, San Diego, CA, USA) was applied to process the raw fluorescent images and the call sequences. The raw reads containing three or more “N” or contaminated by adapter (>15 bp overlap) were removed, and the filtered clean reads (about 1.6 Gb per each sample) were used for further metagenomic analyses. The sequencing data were deposited in the metagenomics RAST server (MG-RAST) [43] under accession number 4494851.3 (sludge treated with 20 mg/L tetracycline) and 4494856.3 (sludge without tetracycline treatment).

### 3.7. Bioinformatics Analysis

After 454 pyrosequencing, all the reads were subjected to the Pyrosequencing Pipeline Initial Process [44] of the Ribosomal Database Project (RDP): (1) To sort the reads to the designated sample based on their nucleotide barcode; (2) To trim off the adapters and barcodes using the default parameters; and (3) To remove sequences containing ambiguous “N” or shorter than 200 bp [45]. Sequences were de-noised using the “pre.cluster” command in the Mothur platform to remove the erroneous sequences due to pyrosequencing errors [46,47]. PCR chimeras were filtered out using Chimera Slayer [48]. The reads marked as chimeras were extracted and submitted to RDP. Those being assigned to any known genus with 90% confidence were integrated with the non-chimera reads [49], to form the “effective sequences”. The effective sequences of each sample were resubmitted to the RDP Classifier [50] to identify the archaeal and bacterial sequences, and the unexpected archaeal sequences were manually removed. In order to study the tetracycline effect on microbial communities, the samples of day 6 were individually selected for pyrosequencing, which generated a total of 42,556 reads for the four samples. As shown in Table 1, low quality reads were filtered using RDP and the effective reads were obtained after trimming the adapters, barcodes and primers. After denoising, filtering out chimeras and removing the archaeal sequences, the minimum number of bacterial sequences in the four samples was 7097. To fairly compare the four samples at the same sequencing depth, the number of the sequences from each sample was normalized to be 7097 for subsequent bioinformatics analyses. Taxonomic assignment of the sequences was separately performed using the RDP’s Classifier. A bootstrap cutoff of 80% was applied to assign the sequences to different taxonomy levels. Richness and diversity indices including OTUs, Chao 1 estimator and Shannon index, as well as rarefaction curves, were calculated using the relevant RDP modules, including Rarefaction and Shannon & Chao1 index.

Illumina sequencing reads were aligned against a self-established database via off-line BLAST to identify ARGs and plasmids in the sludge samples. A protein database of ARGs were created by downloading all sequences in ARDB (7828 sequences) [51]. A read was identified as an ARG according to its best BLAST hit (blastx) if the similarity was above 90% and the alignments was at least 25 amino acids [35]. The nucleotide sequences of plasmids were downloaded from NCBI RefSeq database (2408 plasmid genome sequences). A read was annotated as plasmids if the best BLAST hits (blastn) had a nucleotide sequence similarity >95% over at least 90 bp alignment [35].

## 4. Conclusions

Tetracycline treatment can affect bacterial community structure and increase total abundance and diversity of *tet* genes in the STP sludge, but tends to reduce the abundance of *sul2* predominant in the sludge without tetracycline treatment. Several genera of TRB, including *Sulfuritalea*, *Armatimonas*, *Prosthecobacter*, *Hyphomicrobium*, *Azonexus*, *Longilinea*, *Paracoccus*, *Novosphingobium* and *Rhodobacter* are present in the sludge. Comparatively, antibiotic treatment at subinhibitory concentrations can pose greater effects on the bacterial community composition. The microbial community shift may be responsible for the ARGs distribution patterns variation induced by the tetracycline treatment. As a culture-independent method, pyrosequencing of 16S rRNA gene provides a comprehensive insight into microbial community structure of ARB. Illumina high-throughput sequencing offers enough sequencing depth for metagenomic analysis of ARGs. Combined use of 454 pyrosequencing and Illumina high-throughput sequencing is considered a promising tool for exploration of ARB and ARGs in the environment.

## Supplementary Files

Supplementary File 1 Supplementary Information (PDF, 707 KB)
