# Supplementary material for: A Comprehensive Insight into Tetracycline Resistant Bacteria and Antibiotic Resistance Genes in Activated Sludge Using Next-Generation Sequencing
Source: Int J Mol Sci. 2014 Jun 5;15(6):10083–100. doi: 10.3390/ijms150610083 (PMC4100141; doi:10.3390/ijms150610083)
Supplement: Supplementary File 1 — Supplementary Information (PDF, 707 KB) [file ijms-15-10083-s001.pdf]

Supplementary Information

Table of Contents

Table S1. Matched high-throughput sequencing reads of antibiotic resistance genes (ARGs) in sludge fed with 0 mg/L tetracycline. S1
Table S2. Matched high-throughput sequencing reads of antibiotic resistance genes (ARGs) in sludge fed with 20 mg/L tetracycline. S12
Table S3. Matched high-throughput sequencing reads of plasmids in sludge cultured with 0 mg/L tetracycline. S21
Table S4. Matched high-throughput sequencing reads of plasmids in sludge cultured with 20 mg/L tetracycline. S32
Table S5. PCR primers of the 15 tetracycline resistance genes detected in this study. S40
Figure S1. Occurrence patterns of 11 *tet* genes in activated sludge analyzed by electrophoresis of PCR products. S41

Table S1. Matched high-throughput sequencing reads of antibiotic resistance genes (ARGs) in sludge fed with 0 mg/L tetracycline of Day 6 against antibiotic resistance database (ARDB).

No.	ARDB Accession Number	Identity (%) n	Hit Length (AA) u	E Value t	Number of Reads	ARG Name	Function	Related Antibiotics
1	CAE53425	90.62	25	7.0×10^{-8}	1628	*sul*2	Sulfonamide-resistant dihydropteroate synthase	sulfonamide
2	YP_001969930	90	28	6.0×10^{-9}	182	*sul*2	Sulfonamide-resistant dihydropteroate synthase	sulfonamide
3	YP_002112964	100	27	1.0×10^{-11}	29	*aph33ib*	Aminoglycoside *O*-phosphotransferase	streptomycin
4	YP_001571041	90	28	5.0×10^{-9}	21	*mac*B	Macrolide-specific efflux system	macrolide
5	YP_002029849	90.32	27	2.0×10^{-8}	21	*sme*E	Multidrug resistance efflux pump	fluoroquinolone
6	YP_002890644	90.32	31	3.0×10^{-10}	21	*bac*A	Undecaprenyl pyrophosphate phosphatase	bacitracin
7	CAM88415	100	32	2.0×10^{-12}	13	*aph6id*	Aminoglycoside *O*-phosphotransferase	streptomycin
8	ABG36700	90.62	32	2.0×10^{-12}	12	*sul*1	Sulfonamide-resistant dihydropteroate synthase	sulfonamide
9	YP_001836040	93.94	29	9.0×10^{-11}	12	*erm*B	rRNA adenine *N*-6-methyltransferase	lincosamide, macrolide, streptogramin_b
10	CAC86407	100	32	3.0×10^{-13}	11	*aph7id*	Aminoglycoside *O*-phosphotransferase	streptomycin
11	YP_002894485	93.94	26	4.0×10^{-10}	11	*ant3ia*	Aminoglycoside *O*-nucleotidylyltransferase	spectinomycin, streptomycin

Table S1. *Cont.*

No.	ARDB Accession Number	Identity (%) n	Hit Length (AA) u	E Value t	Number of Reads	ARG Name	Function	Related Antibiotics
12	AAQ92181	90.91	25	2.0×10^{-7}	6	*mex*B	Multidrug resistance efflux pump	aminoglycoside, beta_lactam, fluoroquinolone, tetracycline, tigecycline
13	ACB20261	100	33	7.0×10^{-13}	6	*aph6id*	Aminoglycoside *O*-phosphotransferase	streptomycin
14	BAF36555	100	32	1.0×10^{-11}	6	*sul*1	Sulfonamide-resistant dihydropteroate synthase	sulfonamide
15	CAG34265	90.62	32	4.0×10^{-10}	6	*sme*E	Multidrug resistance efflux pump	fluoroquinolone
16	ZP_04577926	90	30	3.0×10^{-9}	6	*bac*A	Undecaprenyl pyrophosphate phosphatase	bacitracin
17	YP_523088	90.32	27	2.0×10^{-9}	5	*bac*A	Undecaprenyl pyrophosphate phosphatase	bacitracin
18	ABM94007	90.32	31	3.0×10^{-10}	4	*bac*A	Undecaprenyl pyrophosphate phosphatase	bacitracin
19	EEB58431	93.75	32	1.0×10^{-10}	4	*mex*F	Multidrug resistance efflux pump	chloramphenicol, fluoroquinolone
20	EEQ96502	90	30	8.0×10^{-10}	4	*bac*A	Undecaprenyl pyrophosphate phosphatase	bacitracin
21	YP_002006347	90.62	32	5.0×10^{-11}	4	*bac*A	Undecaprenyl pyrophosphate phosphatase	bacitracin
22	YP_586061	90.91	32	4.0×10^{-11}	4	*mex*B	Multidrug resistance efflux pump	aminoglycoside, beta_lactam, fluoroquinolone, tetracycline, tigecycline
23	ZP_03790349	90.91	33	2.0×10^{-7}	4	*ceo*B	Multidrug resistance efflux pump	chloramphenicol
24	AAY92510	90.32	31	1.0×10^{-9}	3	*mex*F	Multidrug resistance efflux pump	chloramphenicol, fluoroquinolone
25	ABQ96629	100	32	5.0×10^{-12}	3	*sul*1	Sulfonamide-resistant dihydropteroate synthase	sulfonamide

Table S1. *Cont.*

No.	ARDB Accession Number	Identity (%) n	Hit Length (AA) u	E Value t	Number of Reads	ARG Name	Function	Related Antibiotics
26	BAH18720	100	33	2.0×10^{-13}	3	*erm*B	rRNA adenine *N*-6-methyltransferase	lincosamide, macrolide, streptogramin_b
27	CAE50480	100	32	2.0×10^{-13}	3	*sul*1	Sulfonamide-resistant dihydropteroate synthase	sulfonamide
28	CAG74079	90.32	31	2.0×10^{-10}	3	*acr*B	Multidrug resistance efflux pump	acriflavin, aminoglycoside, beta_lactam, glycylcycline, macrolide
29	Q82Y49	90.91	30	1.0×10^{-10}	3	*bac*A	Undecaprenyl pyrophosphate phosphatase	bacitracin
30	YP_001187672	90	30	2.0×10^{-9}	3	*mex*F	Multidrug resistance efflux pump	chloramphenicol, fluoroquinolone
31	YP_001477377	90.32	31	4.0×10^{-10}	3	*ros*B	Potassium antiporter	fosmidomycin
32	YP_001908403	90.62	32	6.0×10^{-10}	3	*acr*B	Multidrug resistance efflux pump	acriflavin, aminoglycoside, beta_lactam, glycylcycline, macrolide
33	YP_002800512	90.62	32	3.0×10^{-10}	3	*mex*F	Multidrug resistance efflux pump	chloramphenicol, fluoroquinolone
34	YP_348389	90.32	31	1.0×10^{-10}	3	*mex*F	Multidrug resistance efflux pump	chloramphenicol, fluoroquinolone
35	YP_985461	92.31	26	2.0×10^{-7}	3	*bac*A	Undecaprenyl pyrophosphate phosphatase	bacitracin
36	ZP_01363330	90.91	26	5.0×10^{-8}	3	*mex*B	Multidrug resistance efflux pump	aminoglycoside, beta_lactam, fluoroquinolone, tetracycline, tigecycline

Table S1. *Cont.*

No.	ARDB Accession Number	Identity (%) n	Hit Length (AA) u	E Value t	Number of Reads	ARG Name	Function	Related Antibiotics
37	ABZ01843	100	31	4.0×10^{-12}	2	*tet*G	Major facilitator superfamily transporter, tetracycline efflux pump	tetracycline
38	ACC96132	100	32	6.0×10^{-13}	2	*aad*D	Aminoglycoside *O*-nucleotidylyltransferase	kanamycin, tobramycin
39	ACN73425	96.97	33	3.0×10^{-12}	2	*sul*1	Sulfonamide-resistant dihydropteroate synthase	sulfonamide
40	ACO92337	92.31	26	4.0×10^{-8}	2	*ant2ia*	Aminoglycoside *O*-nucleotidylyltransferase	dibekacin, gentamicin, kanamycin, sisomicin, tobramycin
41	BAC77251	93.55	31	4.0×10^{-11}	2	*acr*B	Multidrug resistance efflux pump	acriflavin, aminoglycoside, beta_lactam, glycylcycline, macrolide
42	CAA70206	100	33	9.0×10^{-14}	2	*erm*C	rRNA adenine *N*-6-methyltransferase	lincosamide, macrolide, streptogramin_b
43	CAI46978	100	32	6.0×10^{-13}	2	*bl2c_ pse3*	Class A beta-lactamase	carbenicillin, penicillin
44	CAJ75670	100	32	7.0×10^{-14}	2	*lnu*A	Lincosamide nucleotidyltransferase	lincomycin
45	CAM12478	96.97	33	6.0×10^{-13}	2	*tet*32	Ribosomal protection protein	tetracycline
46	CAM88409	100	32	7.0×10^{-13}	2	*cml_e3*	Major facilitator superfamily transporter, chloramphenicol efflux pump	chloramphenicol
47	CAY51926	93.75	28	4.0×10^{-8}	2	*mex*W	Multidrug resistance efflux pump	
48	NP_511233	100	32	9.0×10^{-14}	2	*tet*C	Major facilitator superfamily transporter, tetracycline efflux pump	tetracycline

Table S1. *Cont.*

No.	ARDB Accession Number	Identity (%) n	Hit Length (AA) u	E Value t	Number of Reads	ARG Name	Function	Related Antibiotics
49	NP_792892	90.62	32	3.0×10^{-11}	2	*mex*F	Multidrug resistance efflux pump	chloramphenicol, fluoroquinolone
50	NP_862543	100	32	7.0×10^{-14}	2	*erm*T	rRNA adenine *N*-6-methyltransferase	lincosamide, macrolide, streptogramin_b
51	YP_001346277	90.91	25	1.0×10^{-7}	2	*mex*I	Multidrug resistance efflux pump	
52	YP_001348630	90.32	31	5.0×10^{-11}	2	*mex*Y	Multidrug resistance efflux pump	aminoglycoside, glycylcycline
53	YP_001350280	90.91	32	1.0×10^{-10}	2	*mex*W	Multidrug resistance efflux pump	
54	YP_002240037	93.94	33	1.0×10^{-11}	2	*acr*B	Multidrug resistance efflux pump	acriflavin, aminoglycoside, beta_lactam, glycylcycline, macrolide
55	YP_608682	90.62	32	9.0×10^{-11}	2	*mex*D	Multidrug resistance efflux pump	erythromycin, fluoroquinolone, glycylcycline, roxithromycin
56	YP_970399	100	33	1.0×10^{-12}	2	*bac*A	Undecaprenyl pyrophosphate phosphatase	bacitracin
57	ZP_02632674	100	32	7.0×10^{-13}	2	*tet*P(B)	Ribosomal protection protein	tetracycline
58	ZP_03552050	90.91	31	2.0×10^{-11}	2	*bac*A	Undecaprenyl pyrophosphate phosphatase	bacitracin
59	ZP_03752765	96.97	33	8.0×10^{-15}	2	*ant6ia*	Aminoglycoside *O*-nucleotidylyltransferase	streptomycin
60	ZP_04405450	100	33	9.0×10^{-14}	2	*aph6id*	Aminoglycoside *O*-phosphotransferase	streptomycin
61	AAB84282	93.94	33	3.0×10^{-12}	1	*tet*V	Major facilitator superfamily transporter, tetracycline efflux pump	tetracycline

Table S1. *Cont.*

No.	ARDB Accession Number	Identity (%) n	Hit Length (AA) u	E Value t	Number of Reads	ARG Name	Function	Related Antibiotics
62	AAC36915	100	32	3.0×10^{-14}	1	*erm*Q	rRNA adenine *N*-6-methyltransferase	lincosamide, macrolide, streptogramin_b
63	AAK69613	100	32	6.0×10^{-13}	1	*ere*A	Erythromycin esterase	erythromycin
64	AAM08183	93.94	33	3.0×10^{-12}	1	*bl2D_oxa5*	Class D beta-lactamase	cloxacillin, penicillin
65	AAN06707	100	33	4.0×10^{-14}	1	*tet*A	Major facilitator superfamily transporter, tetracycline efflux pump	tetracycline
66	AAO38916	100	33	1.0×10^{-13}	1	*tet*O	Ribosomal protection protein	tetracycline
67	AAQ21216	90.91	33	9.0×10^{-11}	1	*acr*B	Multidrug resistance efflux pump	acriflavin, aminoglycoside, beta_lactam, glycylcycline, macrolide
68	AAR21617	100	32	4.0×10^{-13}	1	*sul*1	Sulfonamide-resistant dihydropteroate synthase	sulfonamide
69	AAR29485	100	33	1.0×10^{-14}	1	*tet*O	Ribosomal protection protein	tetracycline
70	AAS20534	90.91	33	1.0×10^{-11}	1	*bl2D_oxa2*	Class D beta-lactamase	cloxacillin, penicillin
71	AAU89126	100	33	1.0×10^{-14}	1	*aac6ib*	Aminoglycoside *N*-acetyltransferase	amikacin, dibekacin, isepamicin, netilmicin, sisomicin, tobramycin
72	AAY68306	100	33	2.0×10^{-13}	1	*mef*A	Major facilitator superfamily transporter, Macrolide-Lincosamide-Streptogramin B efflux pump	macrolide

Table S1. *Cont.*

No.	ARDB Accession Number	Identity (%) n	Hit Length (AA) u	E Value t	Number of Reads	ARG Name	Function	Related Antibiotics
73	ABG77966	93.94	33	1.0×10^{-11}	1	*acr*B	Multidrug resistance efflux pump	acriflavin, aminoglycoside, beta_lactam, glycylcycline, macrolide
74	ABL95928	96.88	32	2.0×10^{-15}	1	*aac6iia*	Aminoglycoside *N*-acetyltransferase	dibekacin, gentamicin, netilmicin, sisomicin, tobramycin
75	ABN80430	100	32	4.0×10^{-13}	1	*bl2_veb*	Class A beta-lactamase	cephalosporin, penicillin
76	ABO42050	90.62	32	1.0×10^{-12}	1	*ant2ia*	Aminoglycoside *O*-nucleotidylyltransferase	dibekacin, gentamicin, kanamycin, sisomicin, tobramycin
77	ABP57330	100	32	5.0×10^{-15}	1	*ant6ia*	Aminoglycoside *O*-nucleotidylyltransferase	streptomycin
78	ABR13271	90.91	33	6.0×10^{-12}	1	*acr*B	Multidrug resistance efflux pump	acriflavin, aminoglycoside, beta_lactam, glycylcycline, macrolide
79	ABW72062	96.97	33	2.0×10^{-11}	1	*acr*B	Multidrug resistance efflux pump	acriflavin, aminoglycoside, beta_lactam, glycylcycline, macrolide

Table S1. *Cont.*

No.	ARDB Accession Number	Identity (%) n	Hit Length (AA) u	E Value t	Number of Reads	ARG Name	Function	Related Antibiotics
80	ACK77685	90.91	33	2.0×10^{-11}	1	*acr*B	Multidrug resistance efflux pump	acriflavin, aminoglycoside, beta_lactam, glycylcycline, macrolide
81	ACR66841	96.15	26	2.0×10^{-10}	1	*sul*1	Sulfonamide-resistant dihydropteroate synthase	sulfonamide
82	B7LM76	93.94	33	1.0×10^{-14}	1	*arn*A	Bifunctional enzyme that catalyzes the oxidative decarboxylation of UDP-glucuronic acid (UDP-GlcUA) to UDP-4-keto-arabinose (UDP-Ara4O) and the addition of a formyl group to UDP-4-amino-4-deoxy-L-arabinose (UDP-L-Ara4N) to form UDP-L-4-formamido-arabinose (UDP-L-Ara4FN). The modified arabinose is attached to lipid A and is required for resistance to polymyxin and cationic antimicrobial peptides	polymyxin
83	BAB71966	100	33	6.0×10^{-13}	1	*tet*P(A)	Major facilitator superfamily transporter, tetracycline efflux pump	tetracycline
84	CAA70208	100	32	3.0×10^{-14}	1	*erm*C	rRNA adenine *N*-6-methyltransferase	lincosamide, macrolide, streptogramin_b
85	CAJ45367	100	33	3.0×10^{-13}	1	*mef*A	Major facilitator superfamily transporter, Macrolide-Lincosamide-Streptogramin B efflux pump	macrolide
86	CAL44999	100	33	2.0×10^{-15}	1	*lnu*A	Lincosamide nucleotidyltransferase	lincomycin

Table S1. *Cont.*

No.	ARDB Accession Number	Identity (%) n	Hit Length (AA) u	E Value t	Number of Reads	ARG Name	Function	Related Antibiotics
87	NP_348075	93.94	33	6.0×10^{-13}	1	*tet*P(A)	Major facilitator superfamily transporter, tetracycline efflux pump	tetracycline
88	NP_387454	100	33	5.0×10^{-14}	1	*tet*C	Major facilitator superfamily transporter, tetracycline efflux pump	tetracycline
89	Q51429	100	27	4.0×10^{-9}	1	*bl2D_oxa2*	Class D beta-lactamase	cloxacillin,penicillin
90	XP_002333050	96.97	33	6.0×10^{-12}	1	*tet*C	Major facilitator superfamily transporter, tetracycline efflux pump	tetracycline
91	YP_001155044	92.31	26	4.0×10^{-9}	1	*bac*A	Undecaprenyl pyrophosphate phosphatase	bacitracin
92	YP_001350571	90.91	33	7.0×10^{-11}	1	*mex*D	Multidrug resistance efflux pump	erythromycin, fluoroquinolone, glycylcycline, roxithromycin
93	YP_001477378	90.62	32	6.0×10^{-12}	1	*ros*A	Major facilitator superfamily transporter	fosmidomycin
94	YP_001716198	100	33	1.0×10^{-13}	1	*erm*T	rRNA adenine *N*-6-methyltransferase	lincosamide, macrolide, streptogramin_b
95	YP_001749316	90.32	31	2.0×10^{-10}	1	*mex*F	Multidrug resistance efflux pump	chloramphenicol, fluoroquinolone
96	YP_001797193	93.55	31	3.0×10^{-11}	1	*bac*A	Undecaprenyl pyrophosphate phosphatase	bacitracin
97	YP_002030219	93.75	32	8.0×10^{-12}	1	*sme*B	Multidrug resistance efflux pump	fluoroquinolone
98	YP_002081505	90.62	32	4.0×10^{-11}	1	*mex*F	Multidrug resistance efflux pump	chloramphenicol, fluoroquinolone
99	YP_002084975	90.62	32	3.0×10^{-11}	1	*mex*W	Multidrug resistance efflux pump	
100	YP_002260505	90.91	33	4.0×10^{-12}	1	*bac*A	Undecaprenyl pyrophosphate phosphatase	bacitracin
101	YP_002332890	100	33	2.0×10^{-12}	1	*tet*A	Major facilitator superfamily transporter, tetracycline efflux pump	tetracycline

Table S1. *Cont.*

No.	ARDB Accession Number	Identity (%) n	Hit Length (AA) u	E Value t	Number of Reads	ARG Name	Function	Related Antibiotics
102	YP_002847505	93.75	32	2.0×10^{-10}	1	*mdt*F	Multidrug resistance efflux pump	doxorubicin, erythromycin
103	YP_236040	93.75	32	9.0×10^{-11}	1	*mex*F	Multidrug resistance efflux pump	chloramphenicol, fluoroquinolone
104	YP_236080	90.62	32	1.0×10^{-11}	1	*bac*A	Undecaprenyl pyrophosphate phosphatase	bacitracin
105	YP_274481	90.62	32	3.0×10^{-11}	1	*mex*F	Multidrug resistance efflux pump	chloramphenicol, fluoroquinolone
106	YP_316450	90.62	32	9.0×10^{-11}	1	*bac*A	Undecaprenyl pyrophosphate phosphatase	bacitracin
107	YP_550152	96.97	33	7.0×10^{-13}	1	*bac*A	Undecaprenyl pyrophosphate phosphatase	bacitracin
108	YP_746446	90.91	33	6.0×10^{-12}	1	*bac*A	Undecaprenyl pyrophosphate phosphatase	bacitracin
109	YP_932216	90.91	33	9.0×10^{-11}	1	*bac*A	Undecaprenyl pyrophosphate phosphatase	bacitracin
110	YP_981592	96.97	33	1.0×10^{-12}	1	*bac*A	Undecaprenyl pyrophosphate phosphatase	bacitracin
111	ZP_02901439	93.33	30	2.0×10^{-09}	1	*mdt*F	Multidrug resistance efflux pump	doxorubicin, erythromycin
112	ZP_02904980	96.97	33	7.0×10^{-14}	1	*ceo*B	Multidrug resistance efflux pump	chloramphenicol
113	ZP_03075977	90.32	31	9.0×10^{-11}	1	*arn*A	Bifunctional enzyme that catalyzes the oxidative decarboxylation of UDP-glucuronic acid (UDP-GlcUA) to UDP-4-keto-arabinose (UDP-Ara4O) and the addition of a formyl group to UDP-4-amino-4-deoxy-L-arabinose (UDP-L-Ara4N) to form UDP-L-4-formamido-arabinose (UDP-L-Ara4FN). The modified arabinose is attached to lipid A and is required for resistance to polymyxin and cationic antimicrobial peptides	polymyxin

Table S1. *Cont.*

No.	ARDB Accession Number	Identity (%) n	Hit Length (AA) u	E Value t	Number of Reads	ARG Name	Function	Related Antibiotics
114	ZP_03222019	96.15	26	1.0×10^{-7}	1	*acr*B	Multidrug resistance efflux pump	acriflavin, aminoglycoside, beta_lactam, glycylcycline, macrolide
115	ZP_03541894	90.91	33	8.0×10^{-12}	1	*bac*A	Undecaprenyl pyrophosphate phosphatase	bacitracin
116	ZP_03583212	93.75	32	3.0×10^{-10}	1	*ceo*B	Multidrug resistance efflux pump	chloramphenicol
117	ZP_03832747	90.91	33	2.0×10^{-11}	1	*acr*B	Multidrug resistance efflux pump	acriflavin, aminoglycoside, beta_lactam, glycylcycline, macrolide
118	ZP_04612532	90.91	33	6.0×10^{-12}	1	*acr*B	Multidrug resistance efflux pump	acriflavin, aminoglycoside, beta_lactam, glycylcycline, macrolide
119	ZP_04612557	90.32	31	4.0×10^{-10}	1	*ros*B	Potassium antiporter	fosmidomycin
120	ZP_04630103	90.62	32	1.0×10^{-10}	1	*ros*B	Potassium antiporter	fosmidomycin
121	ZP_04631861	90.91	33	7.0×10^{-11}	1	*ros*B	Potassium antiporter	fosmidomycin

Table S2. Matched high-throughput sequencing reads of antibiotic resistance genes (ARGs) in sludge fed with 20 mg/L tetracycline of Day 6 against antibiotic resistance database (ARDB).

No.	ARDB Accession Number	Identity (%) ≥	Hit Length (AA) u	E Value t	Number of Reads	ARG Name	Function	Related Antibiotics
1	CAE53425	96.97	25	8.0×10^{-10}	72	*sul*2	Sulfonamide-resistant dihydropteroate synthase	sulfonamide
2	YP_002029849	90.32	25	1.0×10^{-8}	43	*sme*E	Multidrug resistance efflux pump	fluoroquinolone
3	ABG36700	92.59	27	2.0×10^{-9}	28	*sul*1	Sulfonamide-resistant dihydropteroate synthase	sulfonamide
4	YP_002894485	90.62	26	4.0×10^{-10}	22	*ant3ia*	Aminoglycoside *O*-nucleotidylyltransferase	spectinomycin, streptomycin
5	YP_002890644	90.62	32	1.0×10^{-11}	20	*bac*A	Undecaprenyl pyrophosphate phosphatase	bacitracin
6	YP_001571041	90.32	31	1.0×10^{-9}	14	*mac*B	Macrolide-specific efflux system	macrolide
7	BAF36555	96.88	32	8.0×10^{-12}	13	*sul*1	Sulfonamide-resistant dihydropteroate synthase	sulfonamide
8	EEB58431	90.62	32	4.0×10^{-10}	13	*mex*F	Multidrug resistance efflux pump	chloramphenicol, fluoroquinolone
9	AAR21617	100	32	4.0×10^{-13}	11	*sul*1	Sulfonamide-resistant dihydropteroate synthase	sulfonamide
10	CAG34265	90.62	32	2.0×10^{-10}	11	*sme*E	Multidrug resistance efflux pump	fluoroquinolone
11	YP_586061	90.91	32	2.0×10^{-11}	10	*mex*B	Multidrug resistance efflux pump	aminoglycoside, beta_lactam, fluoroquinolone, tetracycline, tigecycline
12	ABQ96629	100	26	4.0×10^{-9}	9	*sul*1	Sulfonamide-resistant dihydropteroate synthase	sulfonamide
13	YP_001749316	90.91	32	2.0×10^{-11}	9	*mex*F	Multidrug resistance efflux pump	chloramphenicol, fluoroquinolone
14	YP_001797193	90.62	32	5.0×10^{-11}	8	*bac*A	Undecaprenyl pyrophosphate phosphatase	bacitracin
15	ZP_04577926	90.91	31	5.0×10^{-11}	8	*bac*A	Undecaprenyl pyrophosphate phosphatase	bacitracin

Table S2. *Cont.*

No.	ARDB Accession Number	Identity (%) ≥	Hit Length (AA) u	E Value t	Number of Reads	ARG Name	Function	Related Antibiotics
16	ABO42050	100	32	2.0×10^{-12}	7	*ant2ia*	Aminoglycoside *O*-nucleotidylyltransferase	dibekacin, gentamicin, kanamycin, sisomicin, tobramycin
17	CAC41338	100	32	2.0×10^{-12}	7	*tet*A	Major facilitator superfamily transporter, tetracycline efflux pump	tetracycline
18	YP_002081505	90.62	32	8.0×10^{-10}	7	*mex*F	Multidrug resistance efflux pump	chloramphenicol, fluoroquinolone
19	CAE50480	100	32	3.0×10^{-13}	6	*sul*1	Sulfonamide-resistant dihydropteroate synthase	sulfonamide
20	CAM88409	100	32	5.0×10^{-12}	6	*cml_e3*	Major facilitator superfamily transporter, chloramphenicol efflux pump	chloramphenicol
21	Q82Y49	90.91	29	1.0×10^{-9}	6	*bac*A	Undecaprenyl pyrophosphate phosphatase	bacitracin
22	AAS92348	93.94	32	1.0×10^{-12}	5	*bl2D_oxa10*	Class D beta-lactamase	cloxacillin, penicillin
23	ABS19074	96.97	32	7.0×10^{-13}	5	*tet*A	Major facilitator superfamily transporter, tetracycline efflux pump	tetracycline
24	YP_001350280	90	30	6.0×10^{-10}	5	*mex*W	Multidrug resistance efflux pump	
25	YP_001908403	90.91	27	5.0×10^{-9}	5	*acr*B	Multidrug resistance efflux pump	acriflavin, aminoglycoside, beta_lactam, glycylcycline, macrolide
26	YP_002006347	90.62	32	2.0×10^{-11}	5	*bac*A	Undecaprenyl pyrophosphate phosphatase	bacitracin
27	AAF72942	100	32	3.0×10^{-13}	4	*bl2D_oxa10*	Class D beta-lactamase	cloxacillin, penicillin
28	ABL95928	100	32	3.0×10^{-13}	4	*aac6iia*	Aminoglycoside *N*-acetyltransferase	dibekacin, gentamicin, netilmicin, sisomicin, tobramycin
29	ACB20261	100	32	1.0×10^{-13}	4	*aph6id*	Aminoglycoside *O*-phosphotransferase	streptomycin

Table S2. *Cont.*

No.	ARDB Accession Number	Identity (%) ≥	Hit Length (AA) u	E Value t	Number of Reads	ARG Name	Function	Related Antibiotics
30	CAC86407	100	32	1.0×10^{-13}	4	*aph6id*	Aminoglycoside *O*-phosphotransferase	streptomycin
31	CAI46978	100	32	2.0×10^{-12}	4	*bl2c_pse3*	Class A beta-lactamase	carbenicillin, penicillin
32	NP_511233	96.97	32	7.0×10^{-13}	4	*tet*C	Major facilitator superfamily transporter, tetracycline efflux pump	tetracycline
33	NP_598114	90.62	31	4.0×10^{-11}	4	*mex*D	Multidrug resistance efflux pump	erythromycin, fluoroquinolone, glycylcycline, roxithromycin
34	YP_001187667	90.91	32	2.0×10^{-11}	4	*bac*A	Undecaprenyl pyrophosphate phosphatase	bacitracin
35	YP_001969930	100	30	2.0×10^{-11}	4	*sul*2	Sulfonamide-resistant dihydropteroate synthase	sulfonamide
36	YP_002112964	96.97	32	3.0×10^{-13}	4	*aph33ib*	Aminoglycoside *O*-phosphotransferase	streptomycin
37	ZP_01363330	90.62	32	6.0×10^{-10}	4	*mex*B	Multidrug resistance efflux pump	aminoglycoside, beta_lactam, fluoroquinolone, tetracycline, tigecycline
38	AAG05347	90.62	32	4.0×10^{-11}	3	*bac*A	Undecaprenyl pyrophosphate phosphatase	bacitracin
39	ABZ01843	100	33	6.0×10^{-13}	3	*tet*G	Major facilitator superfamily transporter, tetracycline efflux pump	tetracycline
40	BAC77251	90.91	32	9.0×10^{-11}	3	*acr*B	Multidrug resistance efflux pump	acriflavin, aminoglycoside, beta_lactam, glycylcycline, macrolide
41	CAC47932	100	32	9.0×10^{-14}	3	*tet*X	NADP-requiring oxidoreductase, an enzyme that can modify tetracycline	tetracycline

Table S2. *Cont.*

No.	ARDB Accession Number	Identity (%) ≥	Hit Length (AA) u	E Value t	Number of Reads	ARG Name	Function	Related Antibiotics
42	CAG74079	90.62	32	3.0×10^{-10}	3	*acr*B	Multidrug resistance efflux pump	acriflavin, aminoglycoside, beta_lactam, glycylcycline, macrolide
43	P51563	96.3	27	2.0×10^{-8}	3	*tet*G	Major facilitator superfamily transporter, tetracycline efflux pump	tetracycline
44	Q51429	100	30	1.0×10^{-11}	3	*bl2D_oxa2*	Class D beta-lactamase	cloxacillin, penicillin
45	YP_001346277	90.32	31	6.0×10^{-10}	3	*mex*I	Multidrug resistance efflux pump	
46	YP_001477377	90.62	31	9.0×10^{-11}	3	*ros*B	Potassium antiporter	fosmidomycin
47	YP_608682	90.62	32	3.0×10^{-10}	3	*mex*D	Multidrug resistance efflux pump	erythromycin, fluoroquinolone, glycylcycline, roxithromycin
48	ZP_01974653	92.59	26	3.0×10^{-8}	3	*ant3ia*	Aminoglycoside *O*-nucleotidylyltransferase	spectinomycin, streptomycin
49	AAK69613	100	32	3.0×10^{-12}	2	*ere*A	Erythromycin esterase	erythromycin
50	AAN06707	100	33	1.0×10^{-13}	2	*tet*A	Major facilitator superfamily transporter, tetracycline efflux pump	tetracycline
51	AAR29485	100	32	5.0×10^{-12}	2	*tet*O	Ribosomal protection protein	tetracycline
52	AAS20534	90.32	31	2.0×10^{-10}	2	*bl2D_oxa2*	Class D beta-lactamase	cloxacillin, penicillin
53	AAU89126	100	32	1.0×10^{-12}	2	*aac6ib*	Aminoglycoside *N*-acetyltransferase	amikacin, dibekacin, isepamicin, netilmicin, sisomicin, tobramycin
54	AAZ91706	96.97	32	1.0×10^{-11}	2	*ere*A	Erythromycin esterase	erythromycin
55	ABM94007	90.32	31	7.0×10^{-11}	2	*bac*A	Undecaprenyl pyrophosphate phosphatase	bacitracin
56	CAY49157	90.62	32	4.0×10^{-11}	2	*mex*F	Multidrug resistance efflux pump	chloramphenicol, fluoroquinolone

Table S2. *Cont.*

No.	ARDB Accession Number	Identity (%) ≥	Hit Length (AA) u	E Value t	Number of Reads	ARG Name	Function	Related Antibiotics
57	YP_001186705	90.32	31	2.0×10^{-10}	2	*mex*W	Multidrug resistance efflux pump	
58	YP_002030219	90.91	32	7.0×10^{-11}	2	*sme*B	Multidrug resistance efflux pump	fluoroquinolone
59	YP_002260505	93.75	32	6.0×10^{-12}	2	*bac*A	Undecaprenyl pyrophosphate phosphatase	bacitracin
60	YP_523088	90.62	32	2.0×10^{-11}	2	*bac*A	Undecaprenyl pyrophosphate phosphatase	bacitracin
61	YP_746446	90.91	32	3.0×10^{-12}	2	*bac*A	Undecaprenyl pyrophosphate phosphatase	bacitracin
62	ZP_02901439	93.75	30	4.0×10^{-10}	2	*mdt*F	Multidrug resistance efflux pump	doxorubicin, erythromycin
63	ZP_03222019	96.97	33	1.0×10^{-11}	2	*acr*B	Multidrug resistance efflux pump	acriflavin, aminoglycoside, beta_lactam, glycylcycline, macrolide
64	ZP_03552050	90.91	33	2.0×10^{-11}	2	*bac*A	Undecaprenyl pyrophosphate phosphatase	bacitracin
65	AAL51021	96.97	33	2.0×10^{-11}	1	*aac6ib*	Aminoglycoside N-acetyltransferase	amikacin, dibekacin, isepamicin, netilmicin, sisomicin, tobramycin
66	AAP22012	100	32	3.0×10^{-12}	1	*cml_e8*	Major facilitator superfamily transporter, chloramphenicol efflux pump	chloramphenicol
67	AAQ21216	93.94	33	1.0×10^{-11}	1	*acr*B	Multidrug resistance efflux pump	acriflavin, aminoglycoside, beta_lactam, glycylcycline, macrolide
68	AAQ92181	93.75	32	5.0×10^{-11}	1	*mex*B	Multidrug resistance efflux pump	aminoglycoside, beta_lactam, fluoroquinolone, tetracycline, tigecycline
69	ABB92626	100	33	9.0×10^{-16}	1	*cat*B3	Group B chloramphenicol acetyltransferase	chloramphenicol
70	ABW72062	93.75	32	3.0×10^{-11}	1	*acr*B	Multidrug resistance efflux pump	acriflavin, aminoglycoside, beta_lactam, glycylcycline, macrolide

Table S2. *Cont.*

No.	ARDB Accession Number	Identity (%) ≥	Hit Length (AA) u	E Value t	Number of Reads	ARG Name	Function	Related Antibiotics
71	ABY60438	100	33	4.0×10^{-13}	1	*bl2D_oxa10*	Class D beta-lactamase	cloxacillin, penicillin
72	ACI02041	100	26	8.0×10^{-10}	1	*tet*W	Ribosomal protection protein	tetracycline
73	ACK77685	90.91	33	2.0×10^{-11}	1	*acr*B	Multidrug resistance efflux pump	acriflavin, aminoglycoside, beta_lactam, glycylcycline, macrolide
74	ACN65403	100	30	8.0×10^{-12}	1	*sul*1	Sulfonamide-resistant dihydropteroate synthase	sulfonamide
75	ACR66841	100	32	2.0×10^{-12}	1	*sul*1	Sulfonamide-resistant dihydropteroate synthase	sulfonamide
76	CAA24909	100	33	5.0×10^{-16}	1	*tet*A	Major facilitator superfamily transporter, tetracycline efflux pump	tetracycline
77	CAA33123	100	33	3.0×10^{-13}	1	*sul*1	Sulfonamide-resistant dihydropteroate synthase	sulfonamide
78	CAE81273	100	33	2.0×10^{-14}	1	*cat*B3	Group B chloramphenicol acetyltransferase	chloramphenicol
79	CAI57693	96.97	33	6.0×10^{-15}	1	*dfr*A7	Group A drug-insensitive dihydrofolate reductase	trimethoprim
80	CAP69660	100	33	4.0×10^{-13}	1	*bl2D_oxa5*	Class D beta-lactamase	cloxacillin, penicillin
81	CAX33142	100	33	9.0×10^{-14}	1	*bl2D_oxa10*	Class D beta-lactamase	cloxacillin, penicillin
82	CAY51926	93.94	33	2.0×10^{-11}	1	*mex*W	Multidrug resistance efflux pump	
83	EEQ96502	90.91	33	1.0×10^{-11}	1	*bac*A	Undecaprenyl pyrophosphate phosphatase	bacitracin
84	NP_348075	96.3	27	1.0×10^{-8}	1	*tet*P(A)	Major facilitator superfamily transporter, tetracycline efflux pump	tetracycline
85	NP_940742	100	33	5.0×10^{-14}	1	*tet*33	Major facilitator superfamily transporter, tetracycline efflux pump	tetracycline
86	XP_002333050	100	33	3.0×10^{-13}	1	*tet*C	Major facilitator superfamily transporter, tetracycline efflux pump	tetracycline

Table S2. *Cont.*

No.	ARDB Accession Number	Identity (%) ≥	Hit Length (AA) u	E Value t	Number of Reads	ARG Name	Function	Related Antibiotics
87	YP_001096238	96.97	33	5.0×10^{-11}	1	*tet*33	Major facilitator superfamily transporter, tetracycline efflux pump	tetracycline
88	YP_001348630	90.91	33	5.0×10^{-12}	1	*mex*Y	Multidrug resistance efflux pump	aminoglycoside, glycylcycline
89	YP_001350571	90.91	33	1.0×10^{-10}	1	*mex*D	Multidrug resistance efflux pump	erythromycin, fluoroquinolone, glycylcycline, roxithromycin
90	YP_001416709	90.62	32	1.0×10^{-11}	1	*bac*A	Undecaprenyl pyrophosphate phosphatase	bacitracin
91	YP_001438541	90.62	32	4.0×10^{-11}	1	*mac*B	Macrolide-specific efflux system	macrolide
92	YP_001477378	96.88	32	6.0×10^{-12}	1	*ros*A	Major facilitator superfamily transporter	fosmidomycin
93	YP_001747848	90.62	32	6.0×10^{-10}	1	*mex*W	Multidrug resistance efflux pump	
94	YP_002240037	93.75	32	5.0×10^{-11}	1	*acr*B	Multidrug resistance efflux pump	acriflavin, aminoglycoside, beta_lactam, glycylcycline, macrolide
95	YP_002332890	100	32	2.0×10^{-11}	1	*tet*A	Major facilitator superfamily transporter, tetracycline efflux pump	tetracycline
96	YP_002382193	90.91	33	8.0×10^{-12}	1	*mac*B	Macrolide-specific efflux system	macrolide
97	YP_002800512	96.97	33	2.0×10^{-12}	1	*mex*F	Multidrug resistance efflux pump	chloramphenicol, fluoroquinolone
98	YP_002847505	93.94	33	5.0×10^{-11}	1	*mdt*F	Multidrug resistance efflux pump	doxorubicin, erythromycin
99	YP_151445	96.88	32	8.0×10^{-12}	1	*acr*B	Multidrug resistance efflux pump	acriflavin, aminoglycoside, beta_lactam, glycylcycline, macrolide

Table S2. *Cont.*

No.	ARDB Accession Number	Identity (%) ≥	Hit Length (AA) u	E Value t	Number of Reads	ARG Name	Function	Related Antibiotics
100	YP_236040	90.62	32	1.0×10^{-10}	1	*mex*F	Multidrug resistance efflux pump	chloramphenicol, fluoroquinolone
101	YP_274481	90.32	31	1.0×10^{-10}	1	*mex*F	Multidrug resistance efflux pump	chloramphenicol, fluoroquinolone
102	YP_348389	90.62	32	2.0×10^{-11}	1	*mex*F	Multidrug resistance efflux pump	chloramphenicol, fluoroquinolone
103	YP_454364	96.43	28	4.0×10^{-9}	1	*acr*B	Multidrug resistance efflux pump	acriflavin, aminoglycoside, beta_lactam, glycylcycline, macrolide
104	YP_550152	96.97	33	2.0×10^{-12}	1	*bac*A	Undecaprenyl pyrophosphate phosphatase	bacitracin
105	YP_606823	90.32	31	6.0×10^{-10}	1	*mex*W	Multidrug resistance efflux pump	
106	YP_970399	93.94	33	6.0×10^{-12}	1	*bac*A	Undecaprenyl pyrophosphate phosphatase	bacitracin
107	YP_981592	90	30	1.0×10^{-09}	1	*bac*A	Undecaprenyl pyrophosphate phosphatase	bacitracin
108	YP_997055	96.97	33	7.0×10^{-13}	1	*bac*A	Undecaprenyl pyrophosphate phosphatase	bacitracin
109	ZP_02901868	90.91	33	1.0×10^{-12}	1	*arn*A	Bifunctional enzyme that catalyzes the oxidative decarboxylation of UDP-glucuronic acid (UDP-GlcUA) to UDP-4-keto-arabinose (UDP-Ara4O) and the addition of a formyl group to UDP-4-amino-4-deoxy-L-arabinose (UDP-L-Ara4N) to form UDP-L-4-formamido-arabinose (UDP-L-Ara4FN). The modified arabinose is attached to lipid A and is required for resistance to polymyxin and cationic antimicrobial peptides	polymyxin

Table S2. *Cont.*

No.	ARDB Accession Number	Identity (%) ≥	Hit Length (AA) u	E Value t	Number of Reads	ARG Name	Function	Related Antibiotics
110	ZP_03075977	96.97	33	4.0×10^{-13}	1	*arn*A	Bifunctional enzyme that catalyzes the oxidative decarboxylation of UDP-glucuronic acid (UDP-GlcUA) to UDP-4-keto-arabinose (UDP-Ara4O) and the addition of a formyl group to UDP-4-amino-4-deoxy-L-arabinose (UDP-L-Ara4N) to form UDP-L-4-formamido-arabinose (UDP-L-Ara4FN). The modified arabinose is attached to lipid A and is required for resistance to polymyxin and cationic antimicrobial peptides	polymyxin
111	ZP_03583212	100	33	7.0×10^{-13}	1	*ceo*B	Multidrug resistance efflux pump	chloramphenicol
112	ZP_03790349	93.75	32	4.0×10^{-11}	1	*ceo*B	Multidrug resistance efflux pump	chloramphenicol
113	ZP_04405450	96	25	2.0×10^{-10}	1	*aph6id*	Aminoglycoside *O*-phosphotransferase	streptomycin
114	ZP_04612532	90.62	32	2.0×10^{-11}	1	*acr*B	Multidrug resistance efflux pump	acriflavin, aminoglycoside, beta_lactam, glycylcycline, macrolide
115	ZP_04615639	90.32	31	9.0×10^{-11}	1	*ros*B	Potassium antiporter	fosmidomycin

Table S3. Matched high-throughput sequencing reads of plasmids in sludge cultured with 0mg/L tetracycline of Day 6 against NCBI Plasmid Genome Database.

Accession Number	Bacterial Host	Plasmid Name	Identity (%) t	Hit Length (bp)	E Value t	Number of Reads
NC_006994.1	*Pasteurella multocida*	pCCK381	95	90	6.0×10^{-36}	4274
NC_011092.1	*Salmonella enterica*	pCVM19633_110	95	93	5.0×10^{-37}	3920
NC_013365.1	*Escherichia coli*	pO111_1	95	90	6.0×10^{-36}	2409
NC_011602.1	*Escherichia coli*	pE2348-2	95	90	6.0×10^{-36}	1003
NC_014641.1	*Achromobacter xylosoxidans*	pA81	95	90	2.0×10^{-35}	509
NC_003296.1	*Ralstonia solanacearum*	pGMI1000MP	95	90	1.0×10^{-33}	439
NC_013193.1	*Candidatus Accumulibacter phosphatis*	pAph01	95	92	2.0×10^{-35}	331
NC_013509.1	*Edwardsiella tarda*	pEIB202	96.04	92	7.0×10^{-40}	245
NC_002524.1	*Uncultured eubacterium*	pIE1115	98	96	3.0×10^{-43}	236
NC_010394.1	*Mycobacterium abscessus*	unnamed plasmid	95.83	90	5.0×10^{-37}	172
NC_010935.1	*Comamonas testosteroni*	pCNB	95	90	2.0×10^{-36}	166
NC_014911.1	*Alicycliphilus denitrificans*	pALIDE02	95	90	8.0×10^{-35}	162
NC_014211.1	*Nocardiopsis dassonvillei*	pNDAS01	95	90	3.0×10^{-34}	141
NC_010912.1	*Avibacterium paragallinarum*	pYMH5	98	97	3.0×10^{-43}	137
NC_010510.1	*Methylobacterium radiotolerans*	pMRAD01	95	90	3.0×10^{-34}	135
NC_007974.2	*Cupriavidus metallidurans*	megaplasmid	95	90	1.0×10^{-33}	123
NC_014167.1	*Corynebacterium resistens*	pJA144188	98	93	1.0×10^{-42}	120
NC_006823.1	*Azoarcus* sp.	plasmid 1	95	90	3.0×10^{-34}	110
NC_003350.1	*Pseudomonas putida*	pWW0	95	90	2.0×10^{-35}	110
NC_014208.1	*Klebsiella oxytoca*	pKOX105	95	91	3.0×10^{-38}	106
NC_009140.1	*Salmonella enterica*	pSN254	95	92	6.0×10^{-36}	85
NC_004973.1	*Uncultured eubacterium*	pIE1130	95.05	100	3.0×10^{-38}	84
NC_011667.1	*Thauera* sp.	pTha01	95	92	2.0×10^{-36}	82
NC_001740.1		RSF1010	98	96	4.0×10^{-42}	80
NC_014621.1	*Ketogulonicigenium vulgare*	pYP1	95	90	2.0×10^{-36}	79
NC_004604.2	*Bacillus megaterium*	pBM400	95	90	2.0×10^{-35}	75

Table S3. *Cont.*

Accession Number	Bacterial Host	Plasmid Name	Identity (%) t	Hit Length (bp)	E Value t	Number of Reads
NC_013859.1	*Azospirillum* sp.	pAB510e	95	90	8.0×10^{-35}	59
NC_009753.1	*Paracoccus methylutens*	pMTH1	97	93	2.0×10^{-41}	57
NC_008790.1	*Campylobacter jejuni*	pTet	96	97	7.0×10^{-40}	50
NC_008697.1	*Nocardioides* sp.	pNOCA01	95	91	2.0×10^{-35}	44
NC_009429.1	*Rhodobacter sphaeroides*	pRSPA01	95.65	91	2.0×10^{-35}	40
NC_014107.1	*Enterobacter cloacae* subsp.	pECL_A	95	91	3.0×10^{-38}	37
NC_004059.1	*Bacillus thuringiensis*	pUIBI-1	95	90	1.0×10^{-33}	37
NC_014811.1	*Mycobacterium* sp.	pMSPYR101	96.94	92	2.0×10^{-40}	36
NC_008826.1	*Methylibium petroleiphilum*	RPME01	95	92	6.0×10^{-36}	34
NC_014633.1	*Ilyobacter polytropus*	pILYOP01	95	90	8.0×10^{-35}	33
NC_012527.1	*Deinococcus deserti*	plasmid 1	95	92	2.0×10^{-35}	32
NC_008703.1	*Mycobacterium* sp.	pMKMS01	95	90	2.0×10^{-36}	30
NC_012780.1	*Eubacterium eligens*	unnamed	95	92	1.0×10^{-37}	27
NC_006821.1	*Bacillus thuringiensis*	pBMBt1	95.96	98	1.0×10^{-38}	27
NC_014309.1	*Ralstonia solanacearum*	RCFBPv3_mp	95	90	3.0×10^{-34}	26
NC_008308.1	*Sphingomonas* sp.	pCAR3	95	91	2.0×10^{-36}	25
NC_009507.1	*Sphingomonas wittichii*	pSWIT01	95	93	3.0×10^{-38}	24
NC_008704.1	*Mycobacterium* sp.	pMKMS02	95	98	3.0×10^{-38}	22
NC_002637.1	*Mannheimia haemolytica*	pMHSCS1	96	93	7.0×10^{-40}	22
NC_007800.1	*Bibersteinia trehalosi*	pCCK13698	97	99	2.0×10^{-41}	22
NC_007486.1	*Rhodococcus erythropolis*	pREC1	96.67	90	2.0×10^{-35}	22
NC_009339.1	*Mycobacterium gilvum*	pMFLV01	95	93	5.0×10^{-37}	21
NC_007959.1	*Nitrobacter hamburgensis*	plasmid 1	95	95	5.0×10^{-37}	20
NC_011961.1	*Thermomicrobium roseum*	unnamed	95	90	8.0×10^{-35}	20
NC_014908.1	*Alicycliphilus denitrificans*	pALIDE01	95	90	5.0×10^{-37}	20
NC_006824.1	*Azoarcus* sp.	plasmid 2	95	92	1.0×10^{-37}	17
NC_008242.1	*Mesorhizobium* sp.	plasmid 1	95	91	6.0×10^{-36}	17

Table S3. *Cont.*

Accession Number	Bacterial Host	Plasmid Name	Identity (%) t	Hit Length (bp)	E Value t	Number of Reads
NC_014144.1	*Thiomonas* sp.	pTHI	96	94	7.0×10^{-40}	17
NC_009717.1	*Xanthobacter autotrophicus*	pXAUT01	95.65	92	2.0×10^{-35}	17
NC_014801.1	*Campylobacter jejuni*	pTet	97	91	2.0×10^{-41}	17
NC_009426.1	*Novosphingobium aromaticivorans*	pNL1	95	90		16
NC_012586.1	*Sinorhizobium fredii*	pNGR234b	95	91	4.0×10^{-33}	16
NC_013963.1	*Bacillus* sp.	pBS-01	98	100	6.0×10^{-36}	15
NC_013280.1	*Uncultured bacterium*	pTRACA22	96.94	97	3.0×10^{-43}	15
NC_003430.1	*Uncultured bacterium*	pB4	95	99	2.0×10^{-40}	15
NC_008272.1		pKJK5	95	100	3.0×10^{-38}	14
NC_012811.1	*Methylobacterium extorquens*	megaplasmid	95	93	3.0×10^{-38}	14
NC_014155.1	*Thiomonas intermedia*	pTINT02	95	99	6.0×10^{-36}	13
NC_002698.1	*Shigella flexneri*	pWR501	95	96	3.0×10^{-38}	13
NC_010076.1	*Bacillus thuringiensis*	pBtoxis	96	100	3.0×10^{-38}	12
NC_008766.1	*Acidovorax* sp.	pAOVO02	95	95	7.0×10^{-40}	12
NC_008043.1	*Ruegeria* sp.	mega plasmid	95	90	3.0×10^{-38}	12
NC_008712.1	*Arthrobacter aurescens*	TC1	95	91	1.0×10^{-33}	12
NC_011419.1	*Escherichia coli*	pSE11-1	99	100	8.0×10^{-35}	11
NC_010983.1	*Alpha proteobacterium*	pAP3.9	95	99	7.0×10^{-45}	11
NC_010848.1	*Flavobacterium* sp.	pOAD2	96	100	3.0×10^{-38}	11
NC_006143.1	*Aeromonas punctata*	pFBAOT6	98.02	99	7.0×10^{-40}	11
NC_008608.1	*Pelobacter propionicus*	pPRO2	98	100	3.0×10^{-43}	11
NC_010488.1	*Escherichia coli*	pSMS35_130	95	92	3.0×10^{-43}	11
NC_013855.1	*Azospirillum* sp.	pAB510a	95	90	3.00×10^{-38}	11
NC_009739.1	*Pseudomonas aeruginosa*	pMATVIM-7	95	93	3.0×10^{-34}	10
NC_007100.1	*Pseudomonas aeruginosa*	Rms149	96	92	3.0×10^{-38}	10
NC_013857.1	*Azospirillum* sp.	pAB510c	95	92	5.0×10^{-37}	10

Table S3. *Cont.*

Accession Number	Bacterial Host	Plasmid Name	Identity (%) t	Hit Length (bp)	E Value t	Number of Reads
NC_005873.1	*Oligotropha carboxidovorans*	pHCG3	95	94	1.0×10^{-37}	10
NC_002091.1	*Bacillus thuringiensis*	pTX14-1	95	92	5.0×10^{-37}	9
NC_014231.1	*Escherichia coli*	pKC394	98	95	3.0×10^{-43}	9
NC_013191.1	*Candidatus Accumulibacter phosphatis*	pAph03	95	99	3.0×10^{-38}	8
NC_005793.1	*Achromobacter denitrificans*	pEST4011	95	95	3.0×10^{-38}	8
NC_009508.1	*Sphingomonas wittichii*	pSWIT02	95.92	98	3.0×10^{-38}	8
NC_010558.1	*Escherichia coli*	pIP1206	100	100	2.00×10^{-46}	8
NC_006365.1	*Legionella pneumophila*	pLPP	95.74	92	2.0×10^{-36}	8
NC_009669.1	*Ochrobactrum anthropi*	pOANT01	95	97	1.0×10^{-37}	7
NC_010625.1	*Burkholderia phymatum*	pBPHY01	95	93	6.0×10^{-36}	7
NC_007141.1	*Campylobacter jejuni*	pTet	95	97	1.0×10^{-37}	7
NC_010189.1	*Naegleria gruberi*	unnamed	95	100	3.0×10^{-38}	7
NC_008688.1	*Paracoccus denitrificans*	plasmid 1	95	92	2.0×10^{-35}	7
NC_009475.1	*Bradyrhizobium* sp.	pBBta01	95	98	3.0×10^{-38}	7
NC_014007.1	*Sphingobium japonicum*	pCHQ1	95	92	2.0×10^{-35}	7
NC_002679.1	*Mesorhizobium loti*	pMLa	95	95	3.0×10^{-38}	6
NC_005241.1	*Cupriavidus necator*	pHG1	95	94	2.0×10^{-36}	6
NC_003123.1	*Aeromonas salmonicida salmonicida*	pRAS3.1	100	100	2.0×10^{-46}	6
NC_008055.1	*Uncultured bacterium in freshwater*	QKH54	98	100	3.0×10^{-43}	6
NC_004957.1		p121BS	99	100	7.0×10^{-45}	6
NC_002089.1		pIE1107	99	100	7.00×10^{-45}	6
NC_003042.1	*Clostridium perfringens*	pCP13	97	99	2.0×10^{-41}	6
NC_012855.1	*Ralstonia pickettii*	pRp12D01	96	100	7.00×10^{-40}	6
NC_007972.2	*Cupriavidus metallidurans*	pMOL28	95.88	91	3×10^{-38}	6
NC_007960.1	*Nitrobacter hamburgensis*	plasmid 2	95.05	98	3.00×10^{-38}	6
NC_006385.1	*Uncultured bacterium activated sludge*	pRSB101	97	98	2.0×10^{-41}	6
NC_004956.1	*Pseudomonas* sp.	pADP-1	99	97	7.0×10^{-45}	6

Table S3. *Cont.*

Accession Number	Bacterial Host	Plasmid Name	Identity (%) t	Hit Length (bp)	E Value t	Number of Reads
NC_008010.2	*Deinococcus geothermalis*	pDGEO01	95.56	90	1.0×10^{-33}	5
NC_010937.1	*Clostridium perfringens*	pCW3	96	100	7.0×10^{-40}	5
NC_010643.1	*Providencia rettgeri*	R7K	99	100	7.0×10^{-45}	5
NC_013513.1	*Paracoccus aminophilus*	pAMI3	95	100	3.0×10^{-38}	5
NC_014557.1	*Bacillus* sp.	pBS-02	100	100	2.0×10^{-46}	5
NC_003122.1	*Uncultured bacterium in soil*	pSB102	95.65	92	2.00×10^{-35}	5
NC_006363.1	*Nocardia farcinica*	pNF2	97	92	2.0×10^{-41}	5
NC_009957.1	*Dinoroseobacter shibae*	pDSHI03	97.03	100	2×10^{-41}	5
NC_005307.1	*Gordonia westfalica*	pKB1	95.96	99	3.0×10^{-39}	5
NC_012556.1	*Enterobacter cloacae*	pEC-IMPQ	100	92	4.00×10^{-42}	5
NC_007337.1	*Ralstonia eutropha*	plasmid 1	97.96	98	2.00×10^{-41}	5
NC_013278.1	*Uncultured bacterium*	pTRACA18	96.94	98	2.0×10^{-40}	4
NC_010891.1	*Pseudomonas* sp.	pCT14	99	92	4.0×10^{-42}	4
NC_010864.1	*Pediococcus acidilactici*	pEOC01	95	100	3.00×10^{-38}	4
NC_013190.1	*Candidatus Accumulibacter*	pAph02	96	98	7.0×10^{-40}	4
NC_013279.1	*Uncultured bacterium*	pTRACA20	97	99	2.0×10^{-41}	4
NC_010466.1	*Leuconostoc citreum*	pLCK2	95	100	3.00×10^{-38}	4
NC_005707.1	*Bacillus cereus*	pBc10987	95	94	3.0×10^{-38}	4
NC_013783.1	*Lactococcus lactis*	pAR141	97.06	100	4.00×10^{-42}	4
NC_008758.1	*Polaromonas naphthalenivorans*	pPNAP02	95.65	92	2.00×10^{-35}	4
NC_013858.1	*Azospirillum* sp.	pAB510d	95.05	92	3.00×10^{-38}	4
NC_011339.1	*Bacillus cereus*	pH308197_258	100	100	2.0×10^{-46}	3
NC_007352.1	*Staphylococcus saprophyticus*	pSSP2	98.99	99	3.0×10^{-44}	3
NC_003037.1	*Sinorhizobium meliloti*	pSymA	96	97	7.0×10^{-40}	3
NC_011982.1	*Agrobacterium vitis*	pTiS4	96	100	7.0×10^{-40}	3
NC_004945.1	*Corynebacterium glutamicum*	pCG4	99	100	7.0×10^{-45}	3
NC_014154.1	*Thiomonas intermedia*	pTINT01	95.88	97	3.0×10^{-38}	3

Table S3. *Cont.*

Accession Number	Bacterial Host	Plasmid Name	Identity (%) t	Hit Length (bp)	E Value t	Number of Reads
NC_007773.1	*Clostridium perfringens*	pCPF5603	100	100	2.0×10^{-46}	3
NC_010330.1	*Enterococcus faecium*	pRI1	98	100	3.0×10^{-43}	3
NC_010996.1	*Rhizobium etli*	pB	95.65	92	2.00×10^{-35}	3
NC_010529.1	*Cupriavidus taiwanensis*	pRALTA	95.74	91	2.0×10^{-36}	3
NC_012848.1	*Rhizobium leguminosarum*	pR132501	95	92	2.0×10^{-35}	3
NC_008269.1	*Rhodococcus jostii*	pRHL1	95	97	3.0×10^{-38}	3
NC_008765.1	*Acidovorax* sp.	pAOVO01	100	100	2.00×10^{-46}	3
NC_011604.1	*Salmonella enterica*	pWES-1	95.05	90	3.0×10^{-34}	3
NC_006625.1	*Klebsiella pneumoniae*	pK2044	98	100	3.00×10^{-43}	3
NC_009779.1	*Cronobacter sakazakii*	pESA2	98	100	3.00×10^{-43}	3
NC_009129.1	*Corynebacterium* sp.	pLEW279b	98	100	3.00×10^{-43}	3
NC_014726.1	*Enterococcus faecalis*	pTW9	97	100	2.00×10^{-41}	3
NC_014005.1	*Sphingobium japonicum*	pUT1	95.05	99	3.00×10^{-38}	3
NC_006872.1	*Clostridium perfringens*	pBCNF5603	98	100	3.00×10^{-43}	3
NC_007502.1	*Uncultured bacterium in river sediments*	pB8	97	96	6.0×10^{-41}	3
NC_013452.1	*Staphylococcus aureus*	pT181	100	100	2.00×10^{-46}	3
NC_011371.1	*Rhizobium leguminosarum*	pRLG204	95	97	3.0×10^{-38}	3
NC_012849.1	*Ralstonia pickettii*	pRp12D02	95	95	3×10^{-38}	3
NC_003078.1	*Sinorhizobium meliloti*	pSymB	95	96	1.0×10^{-37}	3
NC_006352.1	*Uncultured bacterium activated sludge*	pTB11	95.92	98	1.00×10^{-38}	3
NC_014035.1	*Rhodobacter capsulatus*	pRCB133	95.7	93	6.0×10^{-36}	2
NC_009620.1	*Sinorhizobium medicae*	pSMED01	95	100	3.0×10^{-38}	2
NC_002143.1	*Comamonas testosteroni*	pPT1	98.97	97	3.0×10^{-43}	2
NC_006388.1	*Uncultured bacterium in activated sludge*	pB3	96	100	7.0×10^{-40}	2
NC_009340.1	*Mycobacterium gilvum*	pMFLV02	96	98	7.0×10^{-40}	2
NC_004341.2	*Weissella cibaria*	pKLCB	95.05	101	3.0×10^{-38}	2
NC_009651.1	*Klebsiella pneumoniae*	pKPN5	100	100	2.0×10^{-46}	2

Table S3. *Cont.*

Accession Number	Bacterial Host	Plasmid Name	Identity (%) t	Hit Length (bp)	E Value t	Number of Reads
NC_010938.1	*Tetragenococcus halophilus*	pSKPB18	100	100	2.0×10^{-46}	2
NC_012473.1	*Bacillus cereus*	p03BB102_179	96	100	7.0×10^{-40}	2
NC_008382.1	*Rhizobium leguminosarum bv.*	pRL7	95	98	3.0×10^{-38}	2
NC_011073.1	*Bacteroides fragilis*	pBFP35	100	100	2.0×10^{-46}	2
NC_005566.1	*Staphylococcus epidermidis*	pSK639	98	100	3.0×10^{-43}	2
NC_013176.1	*Pseudomonas putida*	pW2	99	100	2.0×10^{-45}	2
NC_012661.1	*Haemophilus parasuis*	pHN61	100	100	2.0×10^{-46}	2
NC_002114.1	*Nitrosomonas* sp.	pAYL	96	100	7.0×10^{-40}	2
NC_001446.1	*Bacillus thuringiensis*	pTX14-3	95	100	1.00×10^{-37}	2
NC_007771.1	*Staphylococcus chromogenes*	pLNU4	100	100	2.00×10^{-46}	2
NC_015053.1	*Bifidobacterium longum*	p157F-NC1	100	100	2.00×10^{-46}	2
NC_007956.1	*Bacillus subtilis*	pLS30	100	100	2.00×10^{-46}	2
NC_001766.1	*Bacillus subtilis*	pTA1060	98	100	3.00×10^{-43}	2
NC_015187.1	*Acidiphilium multivorum*	pACMV2	97	100	2.00×10^{-41}	2
NC_011887.1	*Methylobacterium nodulans*	pMNOD02	95.56	90	3.00×10^{-34}	2
NC_014558.1	*Lactobacillus plantarum*	pST-III	95	100	3.00×10^{-38}	2
NC_010283.1	*Bacillus thuringiensis*	pFR55	97	100	2.00×10^{-41}	2
NC_010997.1	*Rhizobium etli*	pC	95	100	3.00×10^{-38}	2
NC_008352.1	*Staphylococcus chromogenes*	pLNU8	100	100	2.00×10^{-46}	2
NC_010180.1	*Bacillus weihenstephanensis*	pBWB401	96	98	7.00×10^{-40}	2
NC_006827.2	*Enterococcus faecalis*	pCF10	100	100	2.00×10^{-46}	2
NC_013034.2	*Staphylococcus aureus*	pKKS825	100	100	2.00×10^{-46}	2
NC_008505.1	*Lactococcus lactis*	plasmid 3	96	100	7.00×10^{-40}	2
NC_012521.1	*Rhodococcus opacus*	pROB02	95	100	3.00×10^{-38}	2
NC_010290.1	*Enterococcus faecium*	pJS33	100	100	2.00×10^{-46}	2
NC_005073.1	*Rhodococcus erythropolis*	pBD2	100	100	2.00×10^{-46}	2
NC_011652.1	*Lactobacillus sakei*	pYC2	99	100	7.00×10^{-45}	2

Table S3. *Cont.*

Accession Number	Bacterial Host	Plasmid Name	Identity (%) t	Hit Length (bp)	E Value t	Number of Reads
NC_005570.1	*Yersinia enterocolitica*	p29807	97.03	100	2×10^{-41}	2
NC_011892.1	*Methylobacterium nodulans*	pMNOD01	95	100	3.00×10^{-38}	2
NC_009128.1	*Corynebacterium* sp.	pLEW279a	100	95	1.00×10^{-43}	2
NC_013856.1	*Azospirillum* sp.	pAB510b	95.65	92	2.00×10^{-35}	2
NC_008384.1	*Rhizobium leguminosarum*	pRL11	95.79	95	5.00×10^{-37}	2
NC_005244.2	*Pseudomonas* sp.	pND6-1	96	96	3.00×10^{-39}	2
NC_007770.1	*Staphylococcus haemolyticus*	pLNU3	100	100	2.00×10^{-46}	2
NC_009841.1	*Bacillus thuringiensis*	pBMB67	98	100	3.00×10^{-43}	2
NC_007950.1	*Polaromonas* sp.	plasmid 2	95	100	3.00×10^{-38}	2
NC_010423.1	*Streptococcus pyogenes*	pRW35	99	100	7.00×10^{-45}	2
NC_002575.1	*Agrobacterium rhizogenes*	pRi1724	95	93	2.0×10^{-35}	2
NC_014172.1	*Bacillus thuringiensis*	pBMB171	95.05	100	3×10^{-38}	2
NC_007792.1	*Staphylococcus aureus*	pUSA03	100	100	2.00×10^{-46}	2
NC_008506.1	*Lactococcus lactis*	plasmid 4	98	100	3.00×10^{-43}	2
NC_009794.1	*Citrobacter koseri*	pCKO2	100	100	2.00×10^{-46}	2
NC_014959.1	*Enterococcus faecium*	pS177	95	100	3.00×10^{-38}	2
NC_014475.1	*Enterococcus faecalis*	pWZ1668	100	100	2.00×10^{-46}	2
NC_010802.1	*Burkholderia multivorans*	pTGL1	100	100	2.00×10^{-46}	2
NC_011996.1	*Macrococcus caseolyticus*	pMCCL2	100	100	2.00×10^{-46}	2
NC_009430.1	*Rhodobacter sphaeroides*	pRSPA02	95	100	3.00×10^{-38}	2
NC_005249.1	*Klebsiella pneumoniae*	pLVPK	100	100	2.00×10^{-46}	2
NC_010309.1	*Acinetobacter venetianus*	pAV1	100	98	2.0×10^{-45}	1
NC_007971.2	*Cupriavidus metallidurans*	pMOL30	98.99	99	3.0×10^{-44}	1
NC_012520.1	*Rhodococcus opacus*	pROB01	101	2	7.0×10^{-40}	1
NC_003124.1	*Aeromonas salmonicida*	pRAS3.2	100	100	2.0×10^{-46}	1
NC_008490.1	*Escherichia coli*	pMG828-5	100	100	2.0×10^{-46}	1
NC_008354.1	*Staphylococcus chromogenes*	pLNU9	100	100	2.0×10^{-46}	1

Table S3. *Cont.*

Accession Number	Bacterial Host	Plasmid Name	Identity (%) t	Hit Length (bp)	E Value t	Number of Reads
NC_009132.1	*Escherichia coli*	pLEW517	100	91	2.0×10^{-41}	1
NC_014385.1	*Escherichia coli*	pEC_L46	97.94	97	6.00×10^{-41}	1
NC_009671.1	*Ochrobactrum anthropi*	pOANT03	98.99	99	3.00×10^{-44}	1
NC_004768.1	*Bifidobacterium longum*	pNAC3	96	100	7.00×10^{-40}	1
NC_007791.1	*Staphylococcus aureus*	pUSA02	100	100	2.00×10^{-46}	1
NC_013442.1	*Gordonia bronchialis*	pGBRO01	100	100	2.00×10^{-46}	1
NC_005076.1	*Staphylococcus sciuri*	pSCFS1	100	100	2.00×10^{-46}	1
NC_006979.1	*Streptococcus pyogenes*	pSM19035	100	100	2.00×10^{-46}	1
NC_014164.1	*Lactobacillus acidipiscis*	pLAC1	98	100	3.00×10^{-43}	1
NC_002137.1	*Lactococcus lactis cremoris*	pNZ4000	98	100	3.00×10^{-43}	1
NC_004954.1	*Micrococcus* sp.	pSD10	97	100	2.00×10^{-41}	1
NC_008246.1	*Sphingobium yanoikuyae*	pYAN-1	98	100	3.00×10^{-43}	1
NC_007336.1	*Ralstonia eutropha*	megaplasmid	96	100	7.00×10^{-40}	1
NC_013860.1	*Azospirillum* sp.	pAB510f	95.92	98	1.00×10^{-38}	1
NC_008501.1	*Streptococcus thermophilus*	plasmid 2	95.92	98	3.00×10^{-38}	1
NC_008538.1	*Arthrobacter* sp.	plasmid 2	95.92	98	1.00×10^{-38}	1
NC_011960.1	*Rhodobacter sphaeroides*	pRSKD131B	96.91	97	7.0×10^{-40}	1
NC_008507.1	*Lactococcus lactis*	plasmid 5	99	100	7.00×10^{-45}	1
NC_007763.1	*Rhizobium etli*	p42b	96.04	101	7.00×10^{-40}	1
NC_006427.1	*Enterococcus faecium*	pJB01	99	100	7.00×10^{-45}	1
NC_005088.1	*Delftia acidovorans*	pUO1	100	100	2.00×10^{-46}	1
NC_007103.1	*Bacillus cereus*	pE33L466	98	100	3.00×10^{-43}	1
NC_000914.2	*Sinorhizobium fredii*	pNGR234a	96	100	7.00×10^{-40}	1
NC_004992.1		pCI411	100	100	2.00×10^{-46}	1
NC_005703.1	*Bacillus mycoides*	pBMY1	96.04	101	7.00×10^{-40}	1
NC_015184.1	*Agrobacterium* sp.	pAspH13-3a	96.74	92	5.00×10^{-37}	1
NC_006822.1	*Leuconostoc citreum*	pIH01	100	100	2.0×10^{-46}	1

Table S3. *Cont.*

Accession Number	Bacterial Host	Plasmid Name	Identity (%) t	Hit Length (bp)	E Value t	Number of Reads
NC_013657.1	*Lactococcus lactis*	pKF147A	100	100	2.00×10^{-46}	1
NC_014312.1	*Klebsiella pneumoniae*	pKP048	99	100	7.0×10^{-45}	1
NC_008760.1	*Polaromonas naphthalenivorans*	pPNAP04	96	100	7.00×10^{-40}	1
NC_007768.1	*Staphylococcus chromogenes*	pLNU1	97	100	2.0×10^{-41}	1
NC_012858.1	*Rhizobium leguminosarum*	pR132502	96.84	95	1.00×10^{-38}	1
NC_003922.1	*Xanthomonas axonopodis* pv.	pXAC64	97	100	2.00×10^{-41}	1
NC_007772.1	*Clostridium perfringens*	pCPF4969	100	99	6.0×10^{-46}	1
NC_004566.1	*Lactobacillus fermentum*	pLME300	100	100	2.00×10^{-46}	1
NC_002075.1	*Bacillus subtilis*	p1414	99	100	7.0×10^{-45}	1
NC_010398.1	*Acinetobacter baumannii*	p3ABSDF	99	100	7.00×10^{-45}	1
NC_010401.1	*Acinetobacter baumannii*	p1ABAYE	97	100	2.00×10^{-41}	1
NC_011355.1	*Mycobacterium liflandii*	pMUM002	97	100	2.00×10^{-41}	1
NC_014843.1	*Escherichia coli*	p3521	98	100	3.00×10^{-43}	1
NC_004320.1	*Corynebacterium efficiens*	pCE3	98.97	97	3.00×10^{-43}	1
NC_005023.1	*Acidithiobacillus ferrooxidans*	pTF5	99	100	7.00×10^{-45}	1
NC_007621.1	*Staphylococcus saprophyticus*	pSES22	99	100	7.00×10^{-45}	1
NC_011743.1	*Escherichia fergusonii*	pEFER	100	100	2.00×10^{-46}	1
NC_010540.1	*Lactococcus garvieae*	pKL0018	100	100	2.00×10^{-46}	1
NC_009227.1	*Burkholderia vietnamiensis*	pBVIE02	95.83	96	5.00×10^{-37}	1
NC_004981.2	*Lactococcus lactis*	pCL2.1	95.92	98	1.00×10^{-38}	1
NC_009139.1	*Yersinia ruckeri*	pYR1	100	100	2.0×10^{-46}	1
NC_004532.1	*Lactobacillus reuteri*	pAE78	100	100	2.00×10^{-46}	1
NC_014133.1	*Leuconostoc kimchii*	LkipL4719	100	100	2.00×10^{-46}	1
NC_006826.1	*Sphingomonas xenophaga*	pSx-Qyy	96	100	7.00×10^{-40}	1
NC_000938.1	*Streptococcus thermophilus*	pER36	97	100	2.00×10^{-41}	1
NC_009666.2	*Lactobacillus plantarum*	pM4	99	100	7.00×10^{-45}	1
NC_005703.1	*Bacillus mycoides*	pBMY1	95.05	101	3.00×10^{-38}	1

Table S3. *Cont.*

Accession Number	Bacterial Host	Plasmid Name	Identity (%) t	Hit Length (bp)	E Value t	Number of Reads
NC_010998.1	*Rhizobium etli*	pA	95	100	3.00×10^{-38}	1
NC_010933.1	*Bacillus cereus*	pBC210	99	100	7.00×10^{-45}	1
NC_009621.1	*Sinorhizobium medicae*	pSMED02	95	100	3.00×10^{-38}	1
NC_004847.1	*Lactococcus lactis cremoris*	pHP003	97	100	2.00×10^{-41}	1
NC_002682.1	*Mesorhizobium loti*	pMLb	95.79	95	5.00×10^{-37}	1
NC_011136.1	*Lactobacillus plantarum*	pLR1	99	100	7.00×10^{-45}	1
NC_009435.1	*Lactococcus lactis*	pGdh442	98.99	99	3.00×10^{-44}	1
NC_008385.1	*Burkholderia cepacia*	plasmid 1	100	100	2.00×10^{-46}	1
NC_009083.1	*Acinetobacter baumannii*	pAB1	95.96	99	3.00×10^{-39}	1
NC_010603.1	*Lactobacillus reuteri*	pLR581	95	100	3.00×10^{-38}	1
NC_009349.1	*Aeromonas salmonicida*	plasmid 4	99	100	7.00×10^{-45}	1
NC_011617.1	*Klebsiella pneumoniae*	pKP96	100	100	2.00×10^{-46}	1

Table S4. Matched high-throughput sequencing reads of plasmids in sludge cultured with 20mg/L tetracycline of Day 6 against NCBI Plasmid Genome Database.

Accession Number	Bacterial Host	Plasmid Name	Identity (%) t	Hit lenGTh (bp) n	E Value t	Number of Reads
NC_013193.1	*Candidatus Accumulibacter phosphatis*	pAph01	95	90	1.0×10^{-33}	764
NC_014641.1	*Achromobacter xylosoxidans*	pA81	95.05	91	1.0×10^{-37}	733
NC_003296.1	*Ralstonia solanacearum*	pGMI1000MP	95	90	3.0×10^{-34}	684
NC_014911.1	*Alicycliphilus denitrificans*	pALIDE02	95	93	1.0×10^{-37}	280
NC_007974.2	*Cupriavidus metallidurans*	megaplasmid	95	90	1.0×10^{-33}	229
NC_011092.1	*Salmonella enterica* subsp.	pCVM19633_110	96	90	1.0×10^{-37}	219
NC_006823.1	*Azoarcus* sp.	plasmid 1	95	90	8.0×10^{-35}	219
NC_010510.1	*Methylobacterium radiotolerans*	pMRAD01	95	90	1.0×10^{-33}	215
NC_011667.1	*Thauera* sp.	pTha01	95	90	6.0×10^{-36}	207
NC_013365.1	*Escherichia coli*	pO111_1	95	94	3.0×10^{-38}	203
NC_003350.1	*Pseudomonas putida*	pWW0	95	91	8.0×10^{-35}	203
NC_010394.1	*Mycobacterium abscessus*	unnamed plasmid	95	97	1.0×10^{-37}	179
NC_014167.1	*Corynebacterium resistens*	pJA144188	97	97	2.0×10^{-41}	162
NC_014211.1	*Nocardiopsis dassonvillei* subsp.	pNDAS01	95	90	3.0×10^{-34}	161
NC_014208.1	*Klebsiella oxytoca*	pKOX105	95.1	94	1.0×10^{-38}	148
NC_009140.1	*Salmonella enterica* subsp.	pSN254	95	90	6.0×10^{-36}	133
NC_006994.1	*Pasteurella multocida*	pCCK381	95	92	3.0×10^{-38}	131
NC_014107.1	*Enterobacter cloacae* subsp.	pECL_A	95	90	6.0×10^{-36}	101
NC_013859.1	*Azospirillum* sp.	pAB510e	95	90	3.0×10^{-34}	98
NC_014621.1	*Ketogulonicigenium vulgare*	pYP1	95	90	3.0×10^{-34}	89
NC_009426.1	*Novosphingobium aromaticivorans*	pNL1	95	90	6.0×10^{-36}	76
NC_010935.1	*Comamonas testosteroni*	pCNB	95	95	3.0×10^{-38}	73
NC_008308.1	*Sphingomonas* sp.	pCAR3	95	90	3.0×10^{-34}	72
NC_014908.1	*Alicycliphilus denitrificans*	pALIDE01	95	94	3.0×10^{-38}	64
NC_004604.2	*Bacillus megaterium*	pBM400	95	90	1.0×10^{-33}	62
NC_009429.1	*Rhodobacter sphaeroides*	pRSPA01	95	91	8.0×10^{-35}	62
NC_012527.1	*Deinococcus deserti*	plasmid 1	95	90	$3.0{\times}10^{-34}$	61

Table S4. *Cont.*

Accession Number	Bacterial Host	Plasmid Name	Identity (%) t	Hit Length (bp) n	E Value t	Number of Reads
NC_006143.1	*Aeromonas punctata*	pFBAOT6	96.04	98	7.0×10^{-40}	51
NC_006824.1	*Azoarcus* sp.	plasmid 2	95	97	3.0×10^{-38}	51
NC_002698.1	*Shigella flexneri*	pWR501	95	100	3.0×10^{-38}	45
NC_013190.1	*Candidatus Accumulibacter phosphatis*	pAph02	95	90	6.0×10^{-36}	42
NC_010488.1	*Escherichia coli*	pSMS35_130	96	97	7.0×10^{-40}	42
NC_008826.1	*Methylibium petroleiphilum*	RPME01	95	94	3.0×10^{-38}	41
NC_011602.1	*Escherichia coli*	pE2348-2	99	90	6.0×10^{-41}	40
NC_009508.1	*Sphingomonas wittichii*	pSWIT02	95	97	3.0×10^{-38}	39
NC_008697.1	*Nocardioides* sp.	pNOCA01	95	95	5.0×10^{-37}	39
NC_014811.1	*Mycobacterium* sp.	pMSPYR101	97	90	6.0×10^{-41}	38
NC_014633.1	*Ilyobacter polytropus*	pILYOP01	95	90	3.0×10^{-34}	38
NC_008272.1	*IncP-1*	pKJK5	95	92	1.0×10^{-37}	37
NC_009753.1	*Paracoccus methylutens*	pMTH1	99	100	7.0×10^{-45}	32
NC_008043.1	*Ruegeria* sp.	mega plasmid	95	95	5.0×10^{-37}	32
NC_003430.1	*Uncultured bacterium*	pB4	95	100	3.0×10^{-38}	31
NC_009507.1	*Sphingomonas wittichii*	pSWIT01	98	99	3.0×10^{-43}	31
NC_013857.1	*Azospirillum* sp.	pAB510c	95	92	2.0×10^{-35}	30
NC_008703.1	*Mycobacterium* sp.	pMKMS01	95	91	2.0×10^{-36}	29
NC_003123.1	*Aeromonas salmonicida*	pRAS3.1	96	96	7.0×10^{-40}	26
NC_014144.1	*Thiomonas* sp.	pTHI	95	97	3.0×10^{-38}	26
NC_007100.1	*Pseudomonas aeruginosa*	Rms149	98	97	3.0×10^{-43}	25
NC_011961.1	*Thermomicrobium roseum*	unnamed	95	91	8.0×10^{-35}	25
NC_013509.1	*Edwardsiella tarda*	pEIB202	99	100	7.0×10^{-45}	24
NC_014155.1	*Thiomonas intermedia*	pTINT02	96	90	3.0×10^{-39}	24
NC_012586.1	*Sinorhizobium fredii*	pNGR234b	95	92	5.0×10^{-37}	23
NC_008704.1	*Mycobacterium* sp.	pMKMS02	95	99	3.0×10^{-38}	22
NC_008608.1	*Pelobacter propionicus*	pPRO2	95	97	3.0×10^{-38}	22

Table S4. *Cont.*

Accession Number	Bacterial Host	Plasmid Name	Identity (%) t	Hit Length (bp) n	E Value t	Number of Reads
NC_006352.1	*Uncultured bacterium activated sludge*	pTB11	95	99	3.0×10^{-38}	22
NC_007486.1	*Rhodococcus erythropolis*	pREC1	99	99	7.0×10^{-45}	22
NC_007972.2	*Cupriavidus metallidurans*	pMOL28	96.81	94	3.0×10^{-38}	22
NC_010558.1	*Escherichia coli*	pIP1206	96	93	7.0×10^{-40}	21
NC_009669.1	*Ochrobactrum anthropi*	pOANT01	95	91	6.0×10^{-36}	20
NC_010891.1	*Pseudomonas* sp.	pCT14	96	100	7.0×10^{-40}	19
NC_014154.1	*Thiomonas intermedia*	pTINT01	95	95	5.0×10^{-37}	17
NC_011419.1	*Escherichia coli*	pSE11-1	97.98	99	1.0×10^{-42}	15
NC_009339.1	*Mycobacterium gilvum*	pMFLV01	95	92	5.0×10^{-37}	14
NC_014007.1	*Sphingobium japonicum*	pCHQ1	95	90	3.0×10^{-34}	14
NC_009717.1	*Xanthobacter autotrophicus*	pXAUT01	95	97	3.0×10^{-38}	14
NC_009779.1	*Cronobacter sakazakii*	pESA2	95	99	3.0×10^{-38}	13
NC_014309.1	*Ralstonia solanacearum*	RCFBPv3_mp	95	90	3.0×10^{-34}	12
NC_014231.1	*Escherichia coli*	pKC394	96	99	7.0×10^{-40}	12
NC_003122.1	*Uncultured bacterium in soil*	pSB102	98.97	97	3.0×10^{-43}	12
NC_008712.1	*Arthrobacter aurescens*	TC1	95.65	92	2.0×10^{-35}	11
NC_013855.1	*Azospirillum* sp.	pAB510a	95	90	1.0×10^{-33}	10
NC_008010.2	*Deinococcus geothermalis*	pDGEO01	95.05	91	2.0×10^{-36}	10
NC_007353.2	*Sphingomonas* sp.	pA1	95	100	3.0×10^{-38}	10
NC_005241.1	*Cupriavidus necator*	pHG1	95	90	3.0×10^{-34}	10
NC_013858.1	*Azospirillum* sp.	pAB510d	95	90	3.0×10^{-34}	9
NC_001740.1		RSF1010	98	100	3.0×10^{-43}	9
NC_012811.1	*Methylobacterium extorquens*	megaplasmid	95	92	2.0×10^{-35}	9
NC_008242.1	*Mesorhizobium* sp.	plasmid 1	95	93	1.0×10^{-37}	8
NC_012556.1	*Enterobacter cloacae*	pEC-IMPQ	95	100	3.0×10^{-38}	8
NC_007502.1	*Uncultured bacterium in river sediments*	pB8	97.98	91	7.0×10^{-40}	7
NC_009139.1	*Yersinia ruckeri*	pYR1	96	100	7.0×10^{-40}	7
NC_006388.1	*Uncultured bacterium in activated sludge*	pB3	96.77	93	1.0×10^{-37}	7

Table S4. *Cont.*

Accession Number	Bacterial Host	Plasmid Name	Identity (%) t	Hit Length (bp) n	E Value t	Number of Reads
NC_009475.1	*Bradyrhizobium* sp.	pBBta01	95	98	3.0×10^{-38}	6
NC_008765.1	*Acidovorax* sp.	pAOVO01	95.96	99	3.0×10^{-39}	6
NC_012849.1	*Ralstonia pickettii*	pRp12D02	95.56	90	1.0×10^{-33}	6
NC_013191.1	*Candidatus Accumulibacter phosphatis*	pAph03	97	100	2.0×10^{-41}	6
NC_009739.1	*Pseudomonas aeruginosa*	pMATVIM-7	97	95	2.0×10^{-41}	6
NC_006625.1	*Klebsiella pneumoniae*	pK2044	97	100	2.0×10^{-41}	6
NC_008766.1	*Acidovorax* sp.	pAOVO02	95.05	100	3.0×10^{-38}	6
NC_005873.1	*Oligotropha carboxidovorans*	pHCG3	95	97	3.0×10^{-38}	6
NC_009128.1	*Corynebacterium* sp.	pLEW279a	97	97	2.0×10^{-41}	6
NC_006385.1	*Uncultured bacterium activated sludge*	pRSB101	97	100	2.0×10^{-41}	6
NC_002679.1	*Mesorhizobium loti*	pMLa	95	90	6.0×10^{-36}	6
NC_009621.1	*Sinorhizobium medicae*	pSMED02	95	90	3.0×10^{-34}	6
NC_002524.1	*Uncultured eubacterium*	pIE1115	100	100	2.0×10^{-46}	6
NC_007960.1	*Nitrobacter hamburgensis*	plasmid 2	95	91	8.0×10^{-35}	5
NC_009671.1	*Ochrobactrum anthropi*	pOANT03	95	100	3.0×10^{-38}	5
NC_013176.1	*Pseudomonas putida*	pW2	99	100	7.0×10^{-45}	5
NC_015187.1	*Acidiphilium multivorum*	pACMV2	95	92	2.0×10^{-35}	5
NC_007680.1	*Uncultured bacterium*	pTP6	95	100	3.0×10^{-38}	4
NC_009838.1	*Escherichia coli*	pAPEC-O1-R	100	100	2.0×10^{-46}	4
NC_010912.1	*Avibacterium paragallinarum*	pYMH5	99	100	7.0×10^{-45}	4
NC_009349.1	*Aeromonas salmonicida subsp.*	plasmid 4	100	100	2.0×10^{-46}	4
NC_008688.1	*Paracoccus denitrificans*	plasmid 1	95	100	3.0×10^{-38}	4
NC_011617.1	*Klebsiella pneumoniae*	pKP96	100	100	2.0×10^{-46}	4
NC_004956.1	*Pseudomonas* sp.	pADP-1	99	96	3.0×10^{-44}	4
NC_008055.1	*Uncultured bacterium in freshwater*	QKH54	95.96	94	5.0×10^{-37}	4
NC_012723.1	*Burkholderia glumae*	bglu_1p	95	93	1.0×10^{-37}	4
NC_011892.1	*Methylobacterium nodulans*	pMNOD01	95.74	94	2.0×10^{-36}	4
NC_014035.1	*Rhodobacter capsulatus*	pRCB133	96	97	7.0×10^{-40}	4

Table S4. *Cont.*

Accession Number	Bacterial Host	Plasmid Name	Identity (%) t	Hit lengTh (bp) n	E Value t	Number of Reads
NC_007336.1	*Ralstonia eutropha*	megaplasmid	96	90	6.0×10^{-36}	4
NC_010189.1	*Naegleria gruberi*	unnamed	95	96	1.0×10^{-37}	4
NC_008573.1	*Shewanella* sp.	plasmid 1	99	100	7.0×10^{-45}	3
NC_008758.1	*Polaromonas naphthalenivorans*	pPNAP02	95.88	97	3.0×10^{-38}	3
NC_007800.1	*Bibersteinia trehalosi*	pCCK13698	100	100	2.0×10^{-46}	3
NC_008269.1	*Rhodococcus jostii*	pRHL1	95.65	92	2.0×10^{-35}	3
NC_007763.1	*Rhizobium etli*	p42b	95.79	95	5.0×10^{-37}	3
NC_004771.1	*Pasteurella multocida*	pJR1	97	100	2.0×10^{-41}	3
NC_003078.1	*Sinorhizobium meliloti*	pSymB	95.88	97	3.0×10^{-38}	3
NC_004973.1	*Uncultured eubacterium*	pIE1130	100	100	2.0×10^{-46}	3
NC_007950.1	*Polaromonas* sp.	plasmid 2	95	90	6.0×10^{-36}	3
NC_008357.1	*Pseudomonas aeruginosa*	pBS228	96	100	7.0×10^{-40}	3
NC_009227.1	*Burkholderia vietnamiensis*	pBVIE02	96.91	97	7.0×10^{-40}	3
NC_008244.1	*Mesorhizobium* sp.	plasmid 3	95	100	3.0×10^{-38}	3
NC_005793.1	*Achromobacter denitrificans*	pEST4011	98	100	3.0×10^{-43}	3
NC_005909.1	*Pseudomonas alcaligenes*	pRA2	100	100	2.0×10^{-46}	3
NC_007337.1	*Ralstonia eutropha*	plasmid 1	100	100	2.0×10^{-46}	3
NC_008757.1	*Polaromonas naphthalenivorans*	pPNAP01	95	90	1.0×10^{-37}	3
NC_008385.1	*Burkholderia cepacia*	plasmid 1	95.88	97	3.0×10^{-38}	3
NC_009651.1	*Klebsiella pneumoniae* subsp.	pKPN5	100	100	2.0×10^{-46}	3
NC_003922.1	*Xanthomonas axonopodis* pv.	pXAC64	97.98	99	1.0×10^{-42}	3
NC_009341.1	*Mycobacterium gilvum*	pMFLV03	96	100	7.0×10^{-40}	3
NC_008384.1	*Rhizobium leguminosarum* bv.	pRL11	95.05	98	3.0×10^{-38}	2
NC_011143.1	*Phenylobacterium zucineum*	unnamed	95.92	98	1.0×10^{-38}	2
NC_013856.1	*Azospirillum* sp.	pAB510b	95.05	90	3.0×10^{-34}	2
NC_000914.2	*Sinorhizobium fredii*	pNGR234a	96	100	3.0×10^{-39}	2
NC_010643.1	*Providencia rettgeri*	R7K	99	100	7.0×10^{-45}	2
NC_010529.1	*Cupriavidus taiwanensis*	pRALTA	95	100	3.0×10^{-38}	2

Table S4. *Cont.*

Accession Number	Bacterial Host	Plasmid Name	Identity (%) t	Hit Length (bp) n	E Value t	Number of Reads
NC_010606.1	*Acinetobacter baumannii*	pACICU2	99	100	7.0×10^{-45}	2
NC_003227.1	*Corynebacterium glutamicum*	pTET3	99	100	7.0×10^{-45}	2
NC_008379.1	*Rhizobium leguminosarum* bv.	pRL9	96.84	95	1.0×10^{-38}	2
NC_009957.1	*Dinoroseobacter shibae*	pDSHI03	96	100	7.0×10^{-40}	2
NC_011982.1	*Agrobacterium vitis*	pTiS4	95	100	3.0×10^{-38}	2
NC_010998.1	*Rhizobium etli*	pA	97	100	2.0×10^{-41}	2
NC_014356.1	*Escherichia coli*	pQ7	100	100	2.0×10^{-46}	2
NC_006363.1	*Nocardia farcinica*	pNF2	97	100	2.0×10^{-41}	2
NC_011371.1	*Rhizobium leguminosarum* bv.	pRLG204	96.94	98	2.0×10^{-40}	2
NC_010625.1	*Burkholderia phymatum*	pBPHY01	95	100	1.0×10^{-37}	2
NC_004808.2	*Streptomyces rochei*	pSLA2-L	95.79	95	2.0×10^{-36}	2
NC_013718.1	*Citrobacter rodentium*	pCROD2	100	100	2.0×10^{-46}	2
NC_010727.1	*Methylobacterium populi*	pMPOP01	100	100	2.0×10^{-46}	2
NC_013281.1	*Uncultured bacterium*	pTRACA30	100	100	2.0×10^{-46}	2
NC_003037.1	*Sinorhizobium meliloti*	pSymA	95.65	92	2.0×10^{-35}	2
NC_009620.1	*Sinorhizobium medicae*	pSMED01	95.96	99	3.0×10^{-39}	2
NC_014801.1	*Campylobacter jejuni* subsp.	pTet	100	100	2.0×10^{-46}	2
NC_010865.1	*Sinorhizobium meliloti*	pSmeSM11b	95.05	92	2.0×10^{-36}	2
NC_007491.1	*Rhodococcus erythropolis*	pREL1	97	100	2.0×10^{-41}	2
NC_007959.1	*Nitrobacter hamburgensis*	plasmid 1	96.97	97	7.0×10^{-40}	2
NC_014005.1	*Sphingobium japonicum*	pUT1	100	100	2.0×10^{-46}	2
NC_010996.1	*Rhizobium etli*	pB	96	100	7.0×10^{-40}	2
NC_014022.1	*Yersinia pestis*	pMT1	100	100	2.0×10^{-46}	1
NC_007961.1	*Nitrobacter hamburgensis*	plasmid 3	96.77	93	1.0×10^{-37}	1
NC_012690.1	*Escherichia coli*	peH4H	100	100	2.0×10^{-46}	1
NC_006830.1	*Achromobacter xylosoxidans*	pA81	100	100	2.0×10^{-46}	1
NC_001370.1		pC30il	100	100	2.0×10^{-46}	1
NC_009340.1	*Mycobacterium gilvum*	pMFLV02	95	100	3.0×10^{-38}	1

Table S4. *Cont.*

Accession Number	Bacterial Host	Plasmid Name	Identity (%) t	Hit Length (bp) n	E Value t	Number of Reads
NC_004840.1	*Uncultured bacterium*	pB10	100	100	2.0×10^{-46}	1
NC_003292.1	*Escherichia coli*	R46	100	100	2.0×10^{-46}	1
NC_006821.1	*Bacillus thuringiensis sv darmstadiensis*	pBMBt1	97	100	2.0×10^{-41}	1
NC_010600.1	*Acidithiobacillus caldus*	pTcM1	97	100	2.0×10^{-41}	1
NC_011368.1	*Rhizobium leguminosarum* bv.	pRLG201	96.91	97	7.0×10^{-40}	1
NC_009132.1	*Escherichia coli*	pLEW517	100	95	1.0×10^{-43}	1
NC_005244.2	*Pseudomonas* sp.	pND6-1	95.96	99	3.0×10^{-39}	1
NC_007971.2	*Cupriavidus metallidurans*	pMOL30	95.79	95	2.0×10^{-36}	1
NC_014478.1	*Klebsiella pneumoniae*	unnamed	100	100	2.0×10^{-46}	1
NC_010847.2	*Paracoccus aminophilus*	pAMI2	97.89	95	2.0×10^{-40}	1
NC_008538.1	*Arthrobacter* sp.	plasmid 2	96	100	7.0×10^{-40}	1
NC_010370.1	*Laribacter hongkongensis*	pHLHK22	100	100	2.0×10^{-46}	1
NC_009469.1	*Acidiphilium cryptum*	pACRY03	96.91	97	7.0×10^{-40}	1
NC_007764.1	*Rhizobium etli*	p42c	95	100	3.0×10^{-38}	1
NC_003124.1	*Aeromonas salmonicida*	pRAS3.2	100	100	2.0×10^{-46}	1
NC_012848.1	*Rhizobium leguminosarum* bv.	pR132501	95.6	91	3.0×10^{-34}	1
NC_005088.1	*Delftia acidovorans*	pUO1	100	98	2.0×10^{-45}	1
NC_007765.1	*Rhizobium etli*	p42e	95	100	1.0×10^{-37}	1
NC_006671.1	*Escherichia coli*	pAPEC-O2-R	100	100	2.0×10^{-46}	1
NC_012220.1	*Lactobacillus plantarum*	pLD1	99	100	7.0×10^{-45}	1
NC_014918.1	*Mesorhizobium ciceri*	pMESCI01	96	100	7.0×10^{-40}	1
NC_008459.1	*Bordetella pertussis*	pBP136	97	100	2.0×10^{-41}	1
NC_009427.1	*Novosphingobium aromaticivorans*	pNL2	95	100	1.0×10^{-37}	1
NC_007507.1	*Xanthomonas campestris* pv.	pXCV183	98	100	3.0×10^{-43}	1
NC_013860.1	*Azospirillum* sp.	pAB510f	95	100	3.0×10^{-38}	1
NC_010997.1	*Rhizobium etli*	pC	95.7	93	2.0×10^{-35}	1
NC_004041.2	*Rhizobium etli*	p42d	98.95	95	4.0×10^{-42}	1
NC_008790.1	*Campylobacter jejuni* subsp.	pTet	98	100	3.0×10^{-43}	1

Table S4. *Cont.*

Accession Number	Bacterial Host	Plasmid Name	Identity (%) t	Hit Length (bp) n	E Value t	Number of Reads
NC_012987.1	*Methylobacterium extorquens*	p1METDI	96.04	101	7.0×10^{-40}	1
NC_004059.1	*Bacillus thuringiensis*	pUIBI-1	98	100	3.0×10^{-43}	1
NC_011984.1	*Agrobacterium vitis*	pAtS4c	97.96	98	4.0×10^{-42}	1
NC_010518.1	*Methylobacterium radiotolerans*	pMRAD05	95.79	95	5.0×10^{-37}	1
NC_011960.1	*Rhodobacter sphaeroides*	pRSKD131B	98	100	3.0×10^{-43}	1
NC_005307.1	*Gordonia westfalica*	pKB1	95	100	1.0×10^{-37}	1
NC_007949.1	*Polaromonas* sp.	plasmid 1	95.96	99	3.0×10^{-39}	1
NC_011355.1	*Mycobacterium liflandii*	pMUM002	97	100	2.0×10^{-41}	1
NC_013201.1	*Halomicrobium mukohataei*	pHmuk01	95	100	3.0×10^{-38}	1
NC_006816.1	*Salmonella enterica* subsp.	pU302L	96	100	3.0×10^{-39}	1
NC_009650.1	*Klebsiella pneumoniae* subsp.	pKPN4	100	100	2.0×10^{-46}	1
NC_006628.1	*Laribacter hongkongensis*	pHLHK8	96.88	96	3.0×10^{-39}	1
NC_014615.1	*Escherichia coli*	pETN48	100	100	2.0×10^{-46}	1
NC_011981.1	*Agrobacterium vitis*	pAtS4e	96	100	7.0×10^{-40}	1
NC_004939.1	*Corynebacterium striatum*	pTP10	100	100	2.0×10^{-46}	1
NC_014312.1	*Klebsiella pneumoniae*	pKP048	98.94	94	2.0×10^{-41}	1
NC_011887.1	*Methylobacterium nodulans*	pMNOD02	95.74	94	2.0×10^{-36}	1
NC_009230.1	*Burkholderia vietnamiensis*	pBVIE01	97	100	2.0×10^{-41}	1
NC_005249.1	*Klebsiella pneumoniae*	pLVPK	100	100	2.0×10^{-46}	1
NC_007926.1	*Pseudomonas putida*	NAH7	95.05	101	1.0×10^{-37}	1

Table S5. PCR primers of the 15 tetracycline resistance genes detected in this study.

Gene	Primer(5'-3')	Fragment Size	Annealing Temperature
*tet*A	Forward GCTACATCCTGCTTGCCTTC<br>Reverse CATAGATCGCCGTGAAGAGG	210	55.5
*tet*B	Forward TTGGTTAGGGGCAAGTTTTG<br>Reverse GTAATGGGCCAATAACACCG	659	56
*tet*C	Forward CTTGAGAGCCTTCAACCCAG<br>Reverse ATGGTCGTCATCTACCTGCC	418	55
*tet*D	Forward AAACCATTACGGCATTCTGC<br>Reverse GACCGGATACACCATCCATC	787	56
*tet*E	Forward AAACCACATCCTCCATACGC<br>Reverse AAATAGGCCACAACCGTCAG	278	55
*tet*G	Forward GCTCGGTGGTATCTCTGCTC<br>Reverse AGCAACAGAATCGGGAACAC	468	55
*tet*K	Forward TCGATAGGAACAGCAGTA<br>Reverse CAGCAGATCCTACTCCTT	169	55
*tet*L	Forward TCGTTAGCGTGCTGTCATTC<br>Reverse GTATCCCACCAATGTAGCCG	267	55
*tet*M	Forward GTGGACAAAGGTACAACGAG<br>Reverse CGGTAAAGTTCGTCACACAC	406	55
*tet*O	Forward AACTTAGGCATTCTGGCTCAC<br>Reverse TCCCACTGTTCCATATCGTCA	515	50.3
*tet*S	Forward CATAGACAAGCCGTTGACC<br>Reverse ATGTTTTTGGAACGCCAGAG	667	56
*tet*P(A)	Forward CTTGGATTGCGGAAGAAGAG<br>Reverse ATATGCCCATTTAACCACGC	676	55
*tet*Q	Forward TTATACTTCCTCCGGCATCG<br>Reverse ATCGGTTCGAGAATGTCCAC	904	55
*tet*W *	Forward GAGAGCCTGCTATATGCCAGC<br>Reverse GGGCGTATCCACAATGTTAAC	168	64
*tet*X	Forward CAATAATTGGTGGTGGACCC<br>Reverse TTCTTACCTTGGACATCCCG	468	55

The primers used in this study were designed according to Ng *et al*. (2001) and Lee *et al*. (1993). Ng, L.-K.; Martin, I.; Alfa, M.; Mulvey, M. Multiplex PCR for the detection of tetracycline resistant genes. *Mol. Cell. Probe*. **2001**, *15*, 209–215. * Lee, C.; Langlois, B.E.; Dawson, K.A. Detection of tetracycline resistance determinants in pig isolates from three herds with different histories of antimicrobial exposure. *Appl. Environ. Microbiol*. **1993**, *59*, 1467–1472.

Figure S1. Occurrence patterns of 11 *tet* genes in activated sludge of Day 0 and Day 6 analyzed by electrophoresis of PCR products. M: DNA Marker (DL2000, TaKaRa, Shiga, Japan).

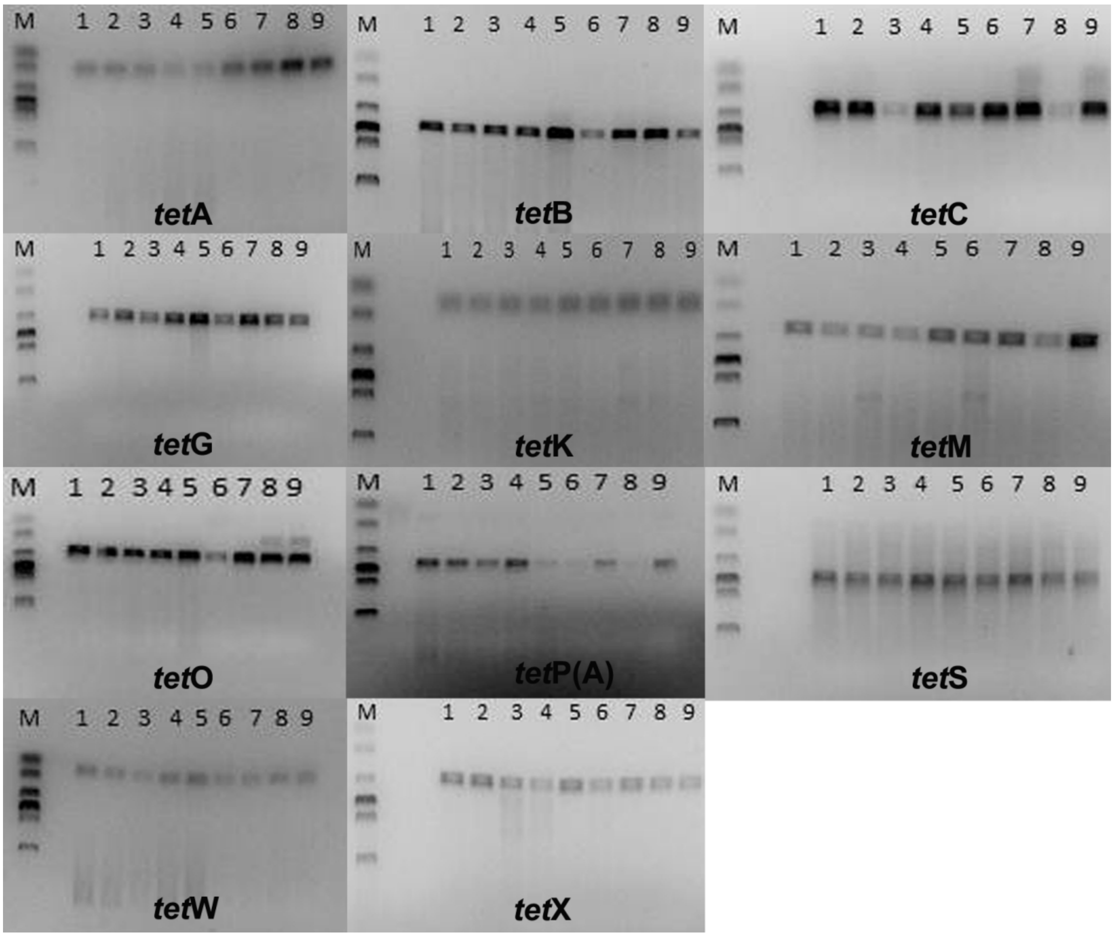

© 2014 by the authors; licensee MDPI, Basel, Switzerland. This article is an open access article distributed under the terms and conditions of the Creative Commons Attribution license (http://creativecommons.org/licenses/by/3.0/).
